# Inactivation of *ca10a* and *ca10b* Genes Leads to Abnormal Embryonic Development and Alters Movement Pattern in Zebrafish

**DOI:** 10.1371/journal.pone.0134263

**Published:** 2015-07-28

**Authors:** Ashok Aspatwar, Martti E. E. Tolvanen, Markus J. T. Ojanen, Harlan R. Barker, Anni K. Saralahti, Carina A. Bäuerlein, Csaba Ortutay, Peiwen Pan, Marianne Kuuslahti, Mataleena Parikka, Mika Rämet, Seppo Parkkila

**Affiliations:** 1 BioMediTech, University of Tampere, Tampere, Finland; 2 School of Medicine, University of Tampere, Tampere, Finland; 3 Department of Information Technology, University of Turku, Turku, Finland; 4 PEDEGO Research Center, and Medical Research Center Oulu, University of Oulu, Oulu, Finland; 5 Department of Children and Adolescents, Oulu University Hospital, Oulu, Finland; 6 Fimlab ltd and Tampere University Hospital, Tampere, Finland; Texas A&M University, UNITED STATES

## Abstract

Carbonic anhydrase related proteins (CARPs) X and XI are highly conserved across species and are predominantly expressed in neural tissues. The biological role of these proteins is still an enigma. Ray-finned fish have lost the *CA11* gene, but instead possess two co-orthologs of *CA10*. We analyzed the expression pattern of zebrafish *ca10a* and *ca10b* genes during embryonic development and in different adult tissues, and studied 61 CARP X/XI-like sequences to evaluate their phylogenetic relationship. Sequence analysis of zebrafish *ca10a* and *ca10b* reveals strongly predicted signal peptides, N-glycosylation sites, and a potential disulfide, all of which are conserved, suggesting that all of CARP X and XI are secretory proteins and potentially dimeric. RT-qPCR showed that zebrafish *ca10a* and *ca10b *genes are expressed in the brain and several other tissues throughout the development of zebrafish. Antisense morpholino mediated knockdown of *ca10a* and *ca10b* showed developmental delay with a high rate of mortality in larvae. Zebrafish morphants showed curved body, pericardial edema, and abnormalities in the head and eye, and there was increased apoptotic cell death in the brain region. Swim pattern showed abnormal movement in morphant zebrafish larvae compared to the wild type larvae. The developmental phenotypes of the *ca10a* and *ca10b* morphants were confirmed by inactivating these genes with the CRISPR/Cas9 system. In conclusion, we introduce a novel zebrafish model to investigate the mechanisms of CARP Xa and CARP Xb functions. Our data indicate that CARP Xa and CARP Xb have important roles in zebrafish development and suppression of *ca10a* and *ca10b* expression in zebrafish larvae leads to a movement disorder.

## Introduction

The α-carbonic anhydrases (α-CA) are zinc-containing metalloenzymes that catalyze the reversible hydration of carbon dioxide (CO_2_ + H_2_O ↔ HCO_3_^-^ + H^+^) [1–3]. In vertebrates there are 17 members in the α-CA gene family (*CA1-17*) which includes 14 catalytically active (CA I, II, III, IV, V, VI, VII, IX, XII, XIII, XIV, XV, XVI, XVII) and 3 inactive (CARP VIII, X, XI) forms [4]. The catalytic inactivity of CARP VIII, X, and XI is due to the lack of one, or more, of the three zinc atom binding histidine residues, which are essential for the classical catalytic activity of CAs [5]. Genes, which code for enzymatically inactive α-CAs, are found in seemingly all metazoan genomes, often in multiple copies. In previous studies of invertebrate CAs, two *CA10*-like genes have been detected in insect genomes [6–8], exemplified by CG32698 and CG1402 in *Drosophila melanogaster*, and two of the six α-CAs in the *Caenorhabditis elegans* genome (*cah-1* and *cah-2*) code for CARP X-like proteins. The *CA10*-like genes in invertebrates are more similar to their vertebrate homologs than are any enzymatically active *CA* genes compared to their vertebrate homologs [7,8].

The expression of *CA10* and *CA11*genes and their protein products has been analyzed in fetal and adult brain in humans, in developing mouse embryos, and in adult mouse tissues [9–12]. In humans the expression of *CA10* and *CA11* has been seen ubiquitously in the central nervous system (CNS), while weak, but significant, signals of the expression were seen in the fetal brain [9]. Similarly, expression studies at the mRNA and protein level showed that the proteins are expressed in all parts of the brain in the adult mouse [6,11]. Developmental expression profiling of *Ca10* and *Ca11* in the brain of mouse embryos showed that *Ca10* mRNA appeared in the middle phase of the gestation, whereas *Ca11* mRNA was seen during early gestational period [11]. In addition, recent studies show that *CA10* is highly expressed in the pineal gland during the nighttime, compared with the daytime, suggesting its involvement in sleep-wake patterns of humans [13].

Previous studies have shown that CARP X and CARP XI play a role in several human diseases such as certain tumors and neurological conditions. For example, human *CA10* sequence contains seven CCG repeats in the 5’-untranslated region followed by two CCG repeats 16 bp downstream of the sequence. The expansion of these trinucleotide repeats lead to various neuropsychiatric diseases in humans [14]. In addition, CARP XI is overexpressed in the gastrointestinal stromal tumors (GISTs), promoting their proliferation and invasion [15]. Finally, three recent expression analyses of *CA11* in transgenic mice with Machado-Joseph disease (MJD), a human patient with Spinocerebellar ataxia type 3 (SCA3), and in cultured neuronal cells producing mutant Ataxin 3 showed an upregulation of CARP XI, suggesting a role for the *CA11* gene in the development of ataxia in humans and mice [16].

The zebrafish has recently emerged as an attractive model organism for studying vertebrate development, as it uniquely combines the advantages of genetic tractability with biologic relevance [17]. Our previous studies showed that there is no *CA11* ortholog in ray-finned fish species, however the *CA10* gene has been duplicated, resulting in genes *ca10a* and *ca10b* [6]. Recently, we have developed an ataxic zebrafish model lacking the *ca8* gene product [18]. The phenotype of these zebrafish resembles that which is observed in human patients with a mutation in the *CA8* gene [18–20]. Our ultimate aim is to find the mechanisms of action and precise physiological roles of *CA10*-like genes, and their protein products, in the brain, especially during embryonic development.

The physiological roles of CARP X and CARP XI have been largely unknown. Furthermore, there have been no systematic studies on the expression pattern of *CA10* and *CA11* genes during embryonic development. Similarly, genetically modified model organisms have not been available to evaluate the function of *CA10* and *CA11* genes. To extend our understanding on the function of the CARP family, we investigated the expression pattern of *ca10a* and *ca10b* genes during embryonic development in zebrafish. In addition, we studied the developmental roles of CARP X and CARP XI by silencing the *ca10a* and *ca10b* genes in zebrafish larvae.

## Materials and Methods

### Sequence analysis

Using an automated pipeline, a total of 83 *CA10* and 54 *CA11* protein sequences, and their corresponding coding regions, were retrieved from vertebrate genomes in the Ensembl database v. 74 and analyzed for completeness. Of these sequences, 46 were identified as complete and correct, and predictions using the Exonerate software package [21] were performed for the remaining incomplete sequences. As a result a total of 16 sequences were manually improved to completeness using the exonerate program. For each analysis, homologous sequences were used as queries to model the gene intron/exon boundaries with full genomes as the search space. Because no bird sequences for *CA11* were found, we performed full-genome scans with Exonerate on all available bird genomes in Ensembl v. 74 (*Ficedula albicollis*, *Gallus gallus*, *Meleagris gallopavo*, and *Taeniopygia guttata*) and one preliminary, unannotated Ensembl genome (*Anas platyrhynchos*). After seeing that the genome of the lamprey in Ensembl (*Petromyzon marinus*) only yielded short sequence fragments of *CA10*, we retrieved the genome of Japanese lamprey (*Lethenteron japonicum*) from the Institute of Molecular and Cell Biology at A*STAR, Singapore (http://jlampreygenome.imcb.a-star.edu.sg/) and performed a full-genome scan to find complete *CA10*-like genes of a jawless vertebrate. Because of the unique position of the jawless vertebrates in evolution, the two nearly complete *L*. *japonicum CA10*-like sequences were also included in further analyses, even though they lack initiation ATG/Met and are slightly shorter than the other sequences. Hence, a total of 57 vertebrate sequences were retrieved for analyses. For comparison purposes, we used two *D*. *melanogaster* CARPs and the cah-2 of *C*. *elegans* directly from the Ensembl and NCBI databases (CARP-A, CG1402: Ensembl FBGN0029962; CARP-B, CG32698: Ensembl FBGN0052698; cah-2: RefSeq NP_495567.3), whereas the existing predicted gene model coding for cah-1 of *C*. *elegans* was revised with the help of Exonerate and support from a fragment sequence FM247165. The full list of sequence names, as they appear in the phylogenetic tree, and their database codes are presented in S1 Table. All multiple sequence alignments were performed with Clustal Omega [22]. To calculate amino acid identity percentages, the number of conserved amino acid residues in each aligned sequence pair was divided by the length of the shorter sequence.

SignalP 4.1 [23] and TargetP 1.1. [24] were used to predict N-terminal secretion signal peptides in the protein sequences (http://www.cbs.dtu.dk/services/). The default cutoff values were used in SignalP (chosen to optimize performance), and predefined cutoffs for specificity >0.95 were used in TargetP. C-terminal glycosylphoshpatidylinositol anchor attachment sites were predicted in the Pred-GPI server [25] (http://gpcr.biocomp.unibo.it/predgpi/) with the general model and taking “Highly probable” and “Probable” predictions (99.5% specificity cutoff) as positive. Potential N-glycosylation sites were identified by the sequence motif (N—not P—S/T—not P).

### Phylogenetics

For phylogenetic analysis, the 57 vertebrate CARP X/XI and Drosophila CARP-B (CG32698) protein sequences were aligned with Clustal Omega. The protein alignment was used as guide in generation of a codon-based coding DNA sequence (CDS) alignment with the Pal2Nal web server [26], with the remove gaps/internal stop codons option. MrBayes [27] was run first to estimate a reliable set of model parameters. To produce our final tree, MrBayes was run for 50,000 generations utilizing the GTR+I (+G) model, with all other parameters as optimized by MrBayes. A 50% majority rule consensus tree was created, rooted by Archaeopteryx [28], with the *Drosophila* sequence as an outgroup. Final trees were drawn aided by the R package Ape [29].

### Protein model

Preliminary protein models of human CARP X were constructed using the I-TASSER server [30,31] at http://zhanglab.ccmb.med.umich.edu/I-TASSER/ by submitting the sequence of residues 22–328. UCSF Chimera 1.8.1 [32] was used for creating model visualizations.

### Zebrafish maintenance and ethics statement

Wild-type zebrafish of the AB and TL strain were maintained at 28.5°C as described previously [33]. The larvae were grown at +28.5°C in embryonic medium (5 mM NaCl, 0.17 mM KCl, 0.33 mM CaCl_2_, 0.33 mM MgSO_4_, and 10–15% Methylene Blue (Sigma-Aldrich)). All the experiments using zebrafish were performed according to the Provincial Government of Western Finland, Province Social and Health Department Tampere Regional Service Unit protocol # LSLH-2007-7254/Ym-23. The care was taken to ameliorate suffering by euthanizing the zebrafish by prolonged immersion in a petridish containing 0.04% tricaine (Sigma-Aldrich).

### Isolation of total RNA and synthesis of cDNA

Total RNA was isolated from whole larvae at different stages of development during 0–168 hpf (0–7 dpf) and from specific organs of the adult zebrafish using the RNeasy mini RNA extraction kit (Qiagen, Hilden, Germany) according to the manufacturer’s instructions. The concentration and purity of total RNA were determined using a NanoDrop Spectrophotometer (NanoDrop 2000, Thermo Scientific). cDNA synthesis was performed using 0.1–5 μg of total RNA and the First Strand cDNA Synthesis kit (Applied Biosystems, Foster City, CA) with random primers and M-MuLV reverse transcriptase according to the protocol recommended by the manufacturer.

### Cloning and sequencing of *ca10a* and *ca10b* genes

*ca10a* and *ca10b* cDNAs were amplified using the primers P1 and P2 (Table 1). The primer P2 adds restriction sites for BamHI and XhoI to the amplification products. The resulting PCR products and pcDNA 3.1 vector were then digested with BamHI and XhoI and ligated with T4 DNA ligase (Promega). The *ca10a* and *ca10b* constructs were then transformed into One Shot TOP10 competent cells (Invitrogen, Espoo, Finland), and the cells were spread on LB plates containing ampicillin (2500μg/ml). The overnight colonies were screened by colony PCR for the presence of the *ca10a* or *ca10b* insert. The pcDNA3.1 plasmid was isolated from the 3-ml overnight culture using Plasmid Maxi Kit (QIAGEN), and sequencing of *ca10a* and *ca10b* was carried out using P2 from the plasmid DNA. The sequences were aligned with ClustalW and compared with cDNA obtained from Ensembl database (Transcript IDs *ca10a* and *ca10b* are ENSDART00000074540 and, ENSDART00000055264 respectively).

**Table 1:** Primer sequences used in the present study.

Gene /Ensembl transcript-ID	Name of the Primer	Upstream primer (5’-3’)	Downstream primer (5’-3’)	Product size (bp)
*ca10a*/ENSDART00000074540	*ca10a*-G-DNA	TCAGGGAAGCCACCCTTGAATGA	AGAGAAGGCCTGGCCATTGAGG	400
	*ca10a*-RT PCR (P1)	ATGGATATAATCTGGGAAATATTTATTATTC	CTATTTCAGCAGCCATTCGTTC	Full Length
	*ca10a*-RT PCR	TCTCTCGGGGCTAAACATTG	TTGACTTAGCAGTCGCATGG	100
	*ca10a*-RT-qPCR	GAAACCAGTCGCATGATTTTTG	TTGCGTTGCCCTGCATT	-
	*ca10a*-T7 (P2) promoter-insertion	TAATACGACTCACTATAGGGATGGATATAATCTGGGAAATATTT	AATCCACCCAAAATCCATGA	Full Length
*ca10b* /ENSDART00000055264	*ca10b* -G-DNA	TAGTGTAAACGGGGCCTTACATTTTA	GATTGTCCTCACTGCCAAAGTG	400
	*ca10b* -RT PCR (P1)	ATGCCGCACGTTTGGGAGTT	TTATTTCAGAAGCCATTCATTCACTC	Full Length
	*ca10b* -RT PCR	TGGGGCTGAATATTGAGGAG	GGCTGGTTTTGACTGAGGAG	100
	*ca10b* -RT-qPCR	CCTGACACCACTGAGGCTCAA	CGGCCGGTGTTGTACATTG	-
	*ca10b* -T7 (P2) promoter-insertion	TAATACGACTCACTATAGGGATGCCGCACGTTTGGGAG	ACGTTTGGGAGTTCGTTCTG	Full Length

### Quantitative analysis of *ca10a* and *ca10b* genes

Real time quantitative PCR (RT-qPCR) primers were designed based on the complete cDNA sequences taken from Ensembl (Transcript IDs as above: using the program Primer Express Software v2.0 (Applied Biosystems)) (Table 1). The RT-qPCR was performed using a SYBR Green PCR Master Mix Kit in an ABI PRISM 7000 Detection System according to the manufacturer's instructions (Applied Biosystems). The PCR conditions consisted of an initial denaturation step at 95°C for 10 min followed by 40 cycles at 95°C for 15 sec (denaturation) and 60°C for 1 min (elongation). The data was analyzed using the ABI PRISM 7000 SDS software (Applied Biosystems). Each PCR reaction was performed in a total reaction volume of 15 μl containing 20ng of cDNA, 1 × Power SYBR green PCR Master Mix (Applied Biosystems, Foster City, CA, USA), and 0.5 μM of each primer. The relative expression of the genes between the studied samples, and internal control gene β-actin were calculated according to Pfaffl's equation [34].

### Knockdown of *ca10a* and *ca10b* genes in zebrafish larvae using antisense morpholino oligonucleotides

To check for the possible polymorphism in genomes at the morpholino target site, we amplified the genomic target region of six adult zebrafish (Table 1) and sequenced the PCR products. The sequenced genomic regions were analyzed for the presence of polymorphism before designing the antisense morpholinos (MOs). Two independent MOs, translation-blocking and splice junction blocking, were designed for the *ca10a* gene, while two splice site blocking MOs, each one targeting a different exon, were designed for the *ca10b* gene. The MOs were designed by Gene Tools (GeneTools LLC, Philomath, OR, USA). As a control, random control (RC) MOs, which did not correspond to any gene in the zebrafish, and *p53*-MOs, which suppress the expression of *p53* mRNA, were used. The control MOs were also obtained from the Gene Tools (Table 2). 1–2 nl of antisense MOs in 0.2 M KCl, 10% phenol red and 10% Rhodamine B (Sigma-Aldrich) was injected into the yolks of one to two-cell stage larvae.

**Table 2:** Morpholino sequences used for knockdown of *ca10a* and *ca10b* genes.

Gene Name	Name of Morpholinos	Morpholino oligonucleotide sequence
*ca10a*	MO1-Translation blocking	5’TGTCTTCATTCCAAGTCCATTGCGC3’
	MO2-Splice site blocking	5’CATCTGTAAGGACAAGCAGAGGTTT3’
*ca10b*	MO1-Splice site blocking	5’ACTGACCTGAAAAACACACCCAAAC3’
	MO2-Splice site blocking	5’GACTGCATCTATGGAAATTCATTAT3’
Radom control (RC)	Control MO	5’CCTCTTACCTCAGTTACAATTTATA3’
*p53*	*p53* MO	5’ GCGCCATTGCTTTGCAAGAATTG 3’

### Phenotypic analysis of morphant and control larvae

The phenotype of the morphant and control embryos (0-5dpf) was analyzed under a light microscope. Approximately 10 to 15 *ca10a* and *ca10b* morphant larvae were screened per set of experiments, with a similar number of control larvae. For the imaging, the larvae were anesthetized using 0.02% Tricaine in embryonic medium and embedded in 17% high molecular weight methylcellulose in 15x30 mm transparent polypropylene Petri dish. The images were taken using a Lumar V1.12 fluorescence stereomicroscope attached to a camera with a 1.5X lens (Carl Zeiss MicroImaging GmbH, Göttingen, Germany). The images were analyzed with AxioVision software versions 4.7 and 4.8.

### Rescue of *ca10a* and *ca10b* morphant zebrafish with mRNA injections

Capped mRNAs encoding full-length sequences of human *CA10* and *CA11* were used for the rescue of *ca10a* and *ca10b* morphant embryos. The human *CA10* and *CA11* clones (*CA10*: IMAGE Id 5276935, CA11: IMAGE Id 3613247) were purchased from the mammalian gene collection (MGC Geneservice Ltd, Cambridge, UK). The coding regions of *CA10* and *CA11* were PCR amplified using the same primers and protocol described previously (Table 1). The restriction sites were added into the PCR product using P2 primers (Table 1) and cloned into a pcDNA3.1 (+) vector. The vector containing the inserts was linearized using KpnI and the capped *CA10* and *CA11* mRNA was transcribed using the mMESSAGE mMACHINE T7 Kit (Ambion), according to the manufacturer’s instructions. For the rescue experiments, two groups of embryos were injected with 300 μM of *CA10*/*CA11* MOs, 300 μM antisense MOs, and 80 pg/embryo of *CA10*/*CA11* capped mRNA.

### Design and production of guide RNAs (gRNAs) for CRISPR/Cas9 mediated genome editing

Target sequences for the *ca10a* and the *ca10b* gRNAs were identified with the online based CRISPR design tool (http://crispr.mit.edu/) and validated with the sgRNA tool of the Casellas laboratory [35], (Retrieved 07:16, May 19, 2015 (GMT)) and BLAST analysis [36]. Initially, three target sequences for *ca10a* and two for *ca10b* were tested (Table 3). The target sequence for *egfp* gRNA was adopted from Jao et al. [37] and gRNAs were produced mostly as described previously [38]. In brief, gRNA oligo (Sigma-Aldrich) and T7 promoter site oligo (Sigma-Aldrich) were annealed and transcribed *in vitro* using the MEGAshortscript T7 Transcription Kit (Ambion Life Technologies, CA, USA). The integrity and size of the produced gRNAs was checked with a 1% agarose Tris-acetate-EDTA (TAE) gel electrophoresis. However, the gRNA design template was slightly modified with two additional guanine nucleotides in the 3´ end of the T7-promoter site (S1 Fig).

**Table 3:** Gene targets and primers for CRISPR/Cas9 mediated knockout studies.

Gene	Targeted exon	Targeted sequence	Primer sequences
*ca10a*	2	TCCACCCAAAATCCATGA	F GAAGCTGTTTGCCAGAAATC
			R ATCCAACACCATCAAAGAAGTC
	2	TACAAAGAAGTTGTTCAG	F GAAGCTGTTTGCCAGAAATC
			R ATCCAACACCATCAAAGAAGTC
	3	ACTGAGGCTCAACACTGG	F CTGCAAATCATCCCTTTGTG
			R GTTCCTCGCATCAAAACACC
*ca10b*	1	AACGAACTCCCAAACGTG	F TCCACGACTCAGCCAACAG
			R GCACTGCGTTATCAGCAAAAG
	3	TTGGGAAGAGACAGTCGC	F CCGCTCTTCCAAACAGATC
			R GTGGATGATTGACAGGGCTC

### gRNA and *cas9* mRNA microinjection and genomic DNA extraction

The gRNAs and the *cas9* mRNA (Invitrogen, Life Technologies) were co-injected into one-cell stage zebrafish embryos with a micro injector (PV830 Pneumatic PicoPump, World Precision Instruments) under a Nikon microscope (SMZ645). The embryos were aligned on 1.2% agarose embryonic medium plates prior to the injection. For genome editing, injection solution of 1 nl contained 270 pg gRNA and 330 pg cas9 mRNA in nuclease-free water. 1.5 ng of phenol red tracer was added to the solution for visualization of the injections. The injection experiments were controlled with un-injected, and gRNA-injected (a mixture of different gRNAs for *ca10a* and *ca10b*, 1280 pg in an injection) embryos. Between 1 and 5 days post fertilization (dpf), zebrafish embryos were visually inspected and imaged with a Lumar V12 microscope (Zeiss, Germany). At 2 and 5 dpf, selected embryos were lysed and genomic DNA isolated from either a single or a pool of embryos (5–10 individuals).

### T7 Endonuclease mutation detection assay

Targeted loci were amplified from genomic zebrafish DNA by PCR using Maxima Hot Start DNA polymerase (Thermo Scientific) according to manufacturer’s instructions. PCR primers (Table 3) were designed to anneal upstream and downstream of the expected cutting site. A previously described T7 endonuclease assay protocol with minor changes was used [35]: 1.5μg of PCR product was annealed in a 20μl reaction of 1x NEBuffer 2 (New England Biolabs, MA, USA) and the annealed sample was incubated with 0.5μl (6 units) of T7 endonuclease I (New England Biolabs) for 30 minutes at 37°C. Obtained products were separated with a 2.5% agarose TAE gel and the band sizes were compared to control samples.

### Histochemical analysis and TUNEL assay

To study the tissue morphology of 5 dpf *ca10a* and *ca10b* morphant larvae a histochemical analysis was performed. Prior to the analysis the larvae were washed with PBS and fixed in 4% paraformaldehyde (PFA) in PBS for 3 hours at room temperature and the fixed larvae were transferred to 70% ethanol and stored at 4°C before being embedded in paraffin. The paraffin embedded samples were sectioned into 5 μm slices for the histochemical staining.

The fixed sections were deparaffinized in xylene, rehydrated in an alcohol series and histologically stained with Mayer's Hematoxylin and Eosin Y (both from Sigma-Aldrich). After dehydration, the slides were mounted with Entellan Neu (Merck; Darmstadt, Germany), examined and photographed using a Nikon Microphot microscope (Nikon Microphot-FXA, Japan).

To detect apoptotic cell death in the *ca10a* and *ca10b* morphants and the control larvae, a TdT-UTP nick end labeling (TUNEL) assay was performed for the prepared slides using the QIA39 FragEL DNA Fragmentation Detection Kit (Merck Chemicals Ltd., Nottingham United Kingdom). Briefly, the deparaffinized sections of the larvae were incubated with the TdT enzyme followed by incubation with anti-digoxigenin. Fluorescence staining was detected and photographed using a Nikon Microphot microscope (Nikon Microphot-FXA, Japan).

### Swim pattern analysis

In order to analyze the minute body movements of the *ca10a* and *ca10b* morphant larvae the swimming pattern of the morphants, uninjected, and the RC MO injected larvae were observed under a microscope. For the analysis, 5 dpf larvae were placed on a Petri dish (5 larvae/dish) containing embryonic medium. The larvae were allowed to settle in the dish for 2 min. The video recording of the swim pattern was done using a Lumar V1.12 fluorescence stereomicroscope (Carl Zeiss MicroImaging GmbH) and AxioVision software versions 4.7 and 4.8. The swimming pattern study of the larvae was repeated a minimum of three times. In addition to these videos, a total of 457 5dpf zebrafish larvae from four groups (*ca10a* morphants, n = 127; *ca10b* morphants, n = 145; RC, n = 101; and uninjected, n = 80) were analyzed for their displacement pattern and distance travelled. In this analysis, the larvae were placed on 90 mm Petri dishes filled with embryonic medium, in groups of 13 to 22, and recorded for 2 minutes. The movements of all the larvae were analyzed using the MtrackJ plugin [39] within the ImageJ program [40]. Distances traveled (cm/1 min) were calculated for each fish.

## Results

### Zebrafish CARP Xa and CARP Xb proteins are similar to human CARP X

As a result of our revised gene prediction analysis within the CARP X/XI family, a total of 881 amino acid positions were improved, and six sequences were improved to completeness (Table 4). The File S1 data includes all novel sequences based on our improved gene models.

**Table 4:** Sequences of CARP X/XI family.

**Focus sequences**			
**Description**	**Complete protein**	**Ensembl Protein ID**	**Ensembl Transcript ID**
Homo sapiens CARP X	x	ENSP00000405388	ENST00000451037
Homo sapiens CARP XI	x	ENSP00000084798	ENST00000084798
Danio rerio CARP Xa	x	ENSDARP00000069028	ENSDART00000074540
Danio rerio CARP Xb	x	ENSDARP00000055263	ENSDART00000055264
**Novel and improved sequences**			
**Description**	**Complete protein**	**Previous Ensembl**	**Changes**
Anas platyrhynchos CARP X	x	ENSAPLP00000003254	Added 75 amino acids in the first 3 exons.
Astyanax mexicanus CARP Xa	x	ENSAMXP00000005715	Replaced 8 amino acids in the 9th exon.
Astyanax mexicanus CARP Xb	x	ENSAMXP00000001335	Added 31 amino acids in the 5th exon.
Choloepus hoffmanni CARP X	x	ENSCHOP00000007972	Added 14 amino acids in the 5th exon.
Dasypus novemcinctus CARP XI	x	ENSDNOP00000030322	Replaced 64 amino acids in 1st and 2nd exons.
Dipodomys ordii CARP X	x	ENSDORP00000010999	Added 22 residues in the 1st exon.
Dipodomys ordii CARP XI	x	ENSDORP00000009964	Replaced 68 amino acids in 6th and 7th exons.
Ficedula albicollis CARP X	x	ENSFALP00000002897	Replaced 15 amino acids in the 4th exon.
Gadus morhua CARP Xb	x	ENSGMOP00000009825	Replaced 25 amino acids in the 6th exon.
Lethenteron japonicum CARP-A	x	**Novel**	Our novel gene model.
Lethenteron japonicum CARP-B	x	**Novel**	Our novel gene model.
Loxodonta africana CARP X	x	ENSLAFP00000013325	Added 7 amino acids in the 9th exon.
Macropus eugenii CARP X	x	ENSMEUP00000011719	Added 1 amino acid in the 1st exon.
Microcebus murinus CARP X	x	ENSMICP00000008888	Replaced 2 amino acids in the 6th exon.
Otolemur garnettii CARP X	x	ENSOGAP00000004458	Replaced 25 amino acids in the 1st exon.
Pteropus vampyrus CARP X	x	ENSPVAP00000013111	Replaced 25 amino acids in the 1st exon.
Sarcophilus harrisii CARP X	x	ENSSHAP00000012943	Added 105 amino acids in the 4th, 5th, and 6th exons.
Tursiops truncatus CARP X	x	ENSTTRP00000001587	Replaced 26 amino acids in the 1st exon.

**Fig 1:**
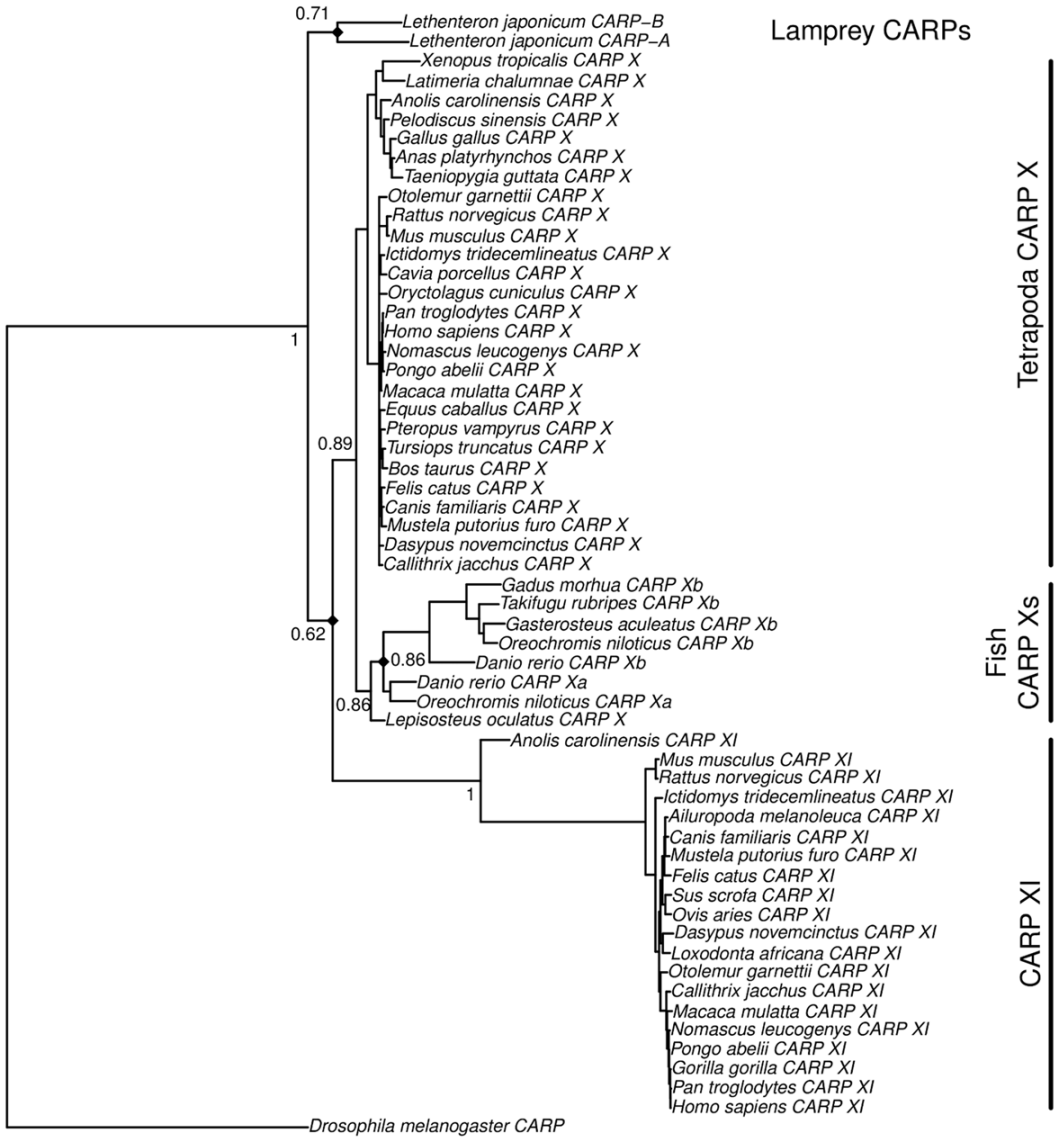
Bayesian phylogenetic tree of CARP sequences. Numbers at the nodes indicate posterior probabilities, and branch lengths are proportional to distances. A table of full details of the sequences is given as S1 Table.

**Fig 2:**
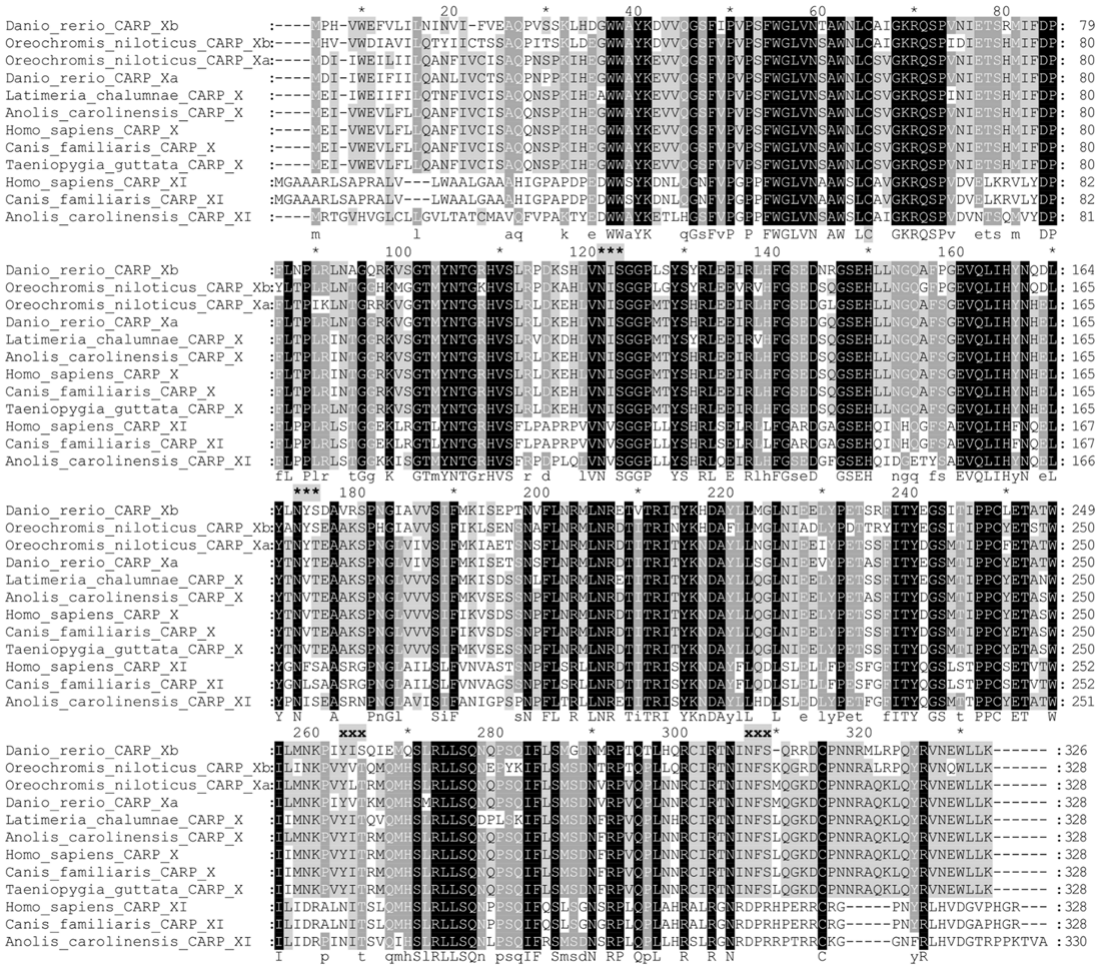
Multiple sequence alignment of selected CARP X-like protein sequences. Groups of three asterisks (***) above the sequences indicate conserved N-glycosylation motifs in all CARP X-like proteins, and letters ‘xxx’ above the sequences indicate N-glycosylation motifs which are only found in either CARP X or CARP XI sequences (NFS and NIT, respectively).

Zebrafish and other ray-finned fishes have two *CA10* orthologs, *ca10a* and *ca10b*, as reported earlier [6]. *ca10a* is highly similar to mammalian *CA10* (90% identity to human CARP X at protein level), whereas *ca10b* is slightly more diverged, yet highly similar (75% identity between human CARP X and zebrafish CARP Xb) (Table 5). In addition, the phylogenetic branching pattern (Fig 1) and the appearance of the alignment (Fig 2) clearly support the notion that fish CARP Xa and Xb are closer to tetrapod CARP X than to CARP XI. The position of the two CARPs of lamprey (*Lethenteron*) indicates that they have duplicated from a single ancestor in the cyclostome lineage, and that the duplication to CARP X and CARP XI took place only in the jawed vertebrates.

**Table 5:** Comparisons of CARP X-like proteins as identity percentages between aligned protein sequences.

	Human CARP X	Human CARP XI	Zebrafish CARP Xa	Zebrafish CARP Xb
**Human CARP X**	100	50	90	75
**Human CARP XI**		100	51	47
**Zebrafish CARP Xa**			100	76
**Zebrafish CARP Xb**				100

It is notable that most vertebrates and even many insects and nematodes have two genes that code for CARP X-like acatalytic proteins, resulting from lineage-specific duplications. However, in case of birds, we could not detect even fragments of genes like *CA11* or *ca10b*, not in database searches nor in Exonerate scans of five bird genomes. In addition to *CA8*, the only gene coding for CARPs in birds is *CA10*, present as one copy per genome.

### CARP X-like proteins have features of secretory proteins conserved from primitive invertebrates

A signal peptide is predicted to be present in 60 of 61 CARP X-like protein sequences by TargetP or in 59 of 61 sequences by SignalP. If we exclude the two lamprey sequences, which lack initial methionine and part of the first exon, we get predicted secretion in all sequences, with reliability classes from 1 to 3 (1 = best, 5 = worst) by TargetP and by SignalP. All tetrapod CARP X sequences, and most fish CARP Xa/Xb sequences, show signal peptide predictions with reliability class 1. The predicted signal peptide cleavage sites in human CARP X and XI, and in zebrafish CARP Xa and CARP Xb coincide with the end of the sequence coded by the first exon in each gene. No glycosyl-phosphatidylinositol anchor attachment sites were predicted for any of the sequences.

**Fig 3:**
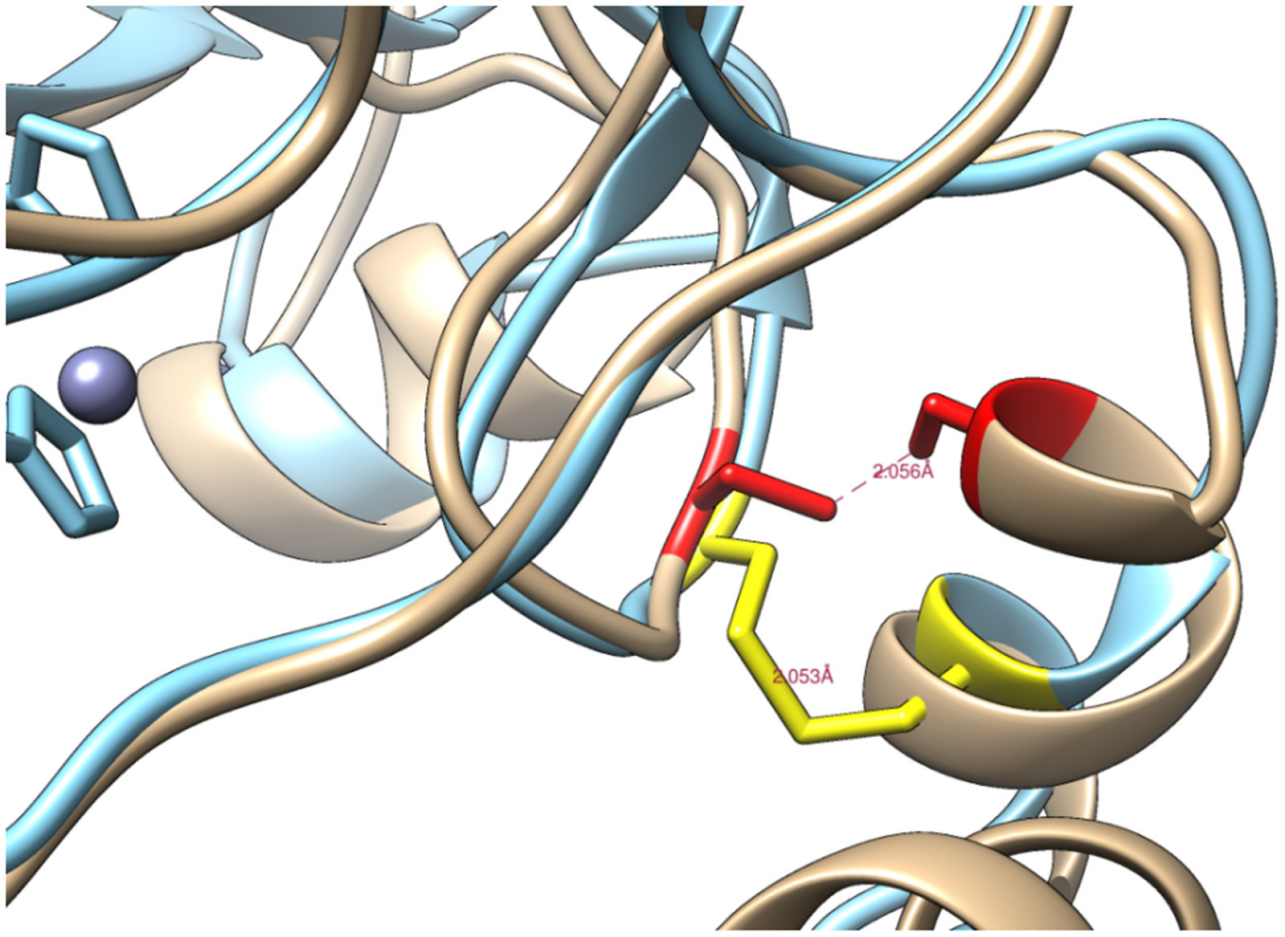
Preliminary model of human CARP X. The model showing a disulfide bridge between conserved cysteines C60 and C223 (marked in red).

All 61 CARP protein sequences in our study possess three conserved cysteines, corresponding to the positions 60, 244, and 310 in human CARP X. In addition, there is a fourth cysteine residue (296 in human CARP X) which is conserved in CARP X in tetrapods, coelacanth (*Latimeria*), the CARPs in lamprey, and CARP Xa and Xb. However, the fourth cysteine residue is not found in CARP XI or in the invertebrate CARP X-like proteins. In addition, there are several non-conserved cysteines in the predicted signal peptide regions. The conserved cysteines C60 and C223 are predicted to be within the CA domain. Their positions in the sequence alignment, and preliminary protein model correspond approximately to the position of the conserved disulfide seen in the structures of CA IV, VI, IX, XII, and XIV, and in sequence alignments of CA XV and XVII [4], but which is absent from other, intracellular CA isoforms (Fig 3) A disulfide with a reasonable geometry could be formed easily, even though the model displaces the first cysteine by one turn of a helix relative to the position seen in other CAs.

**Fig 4:**
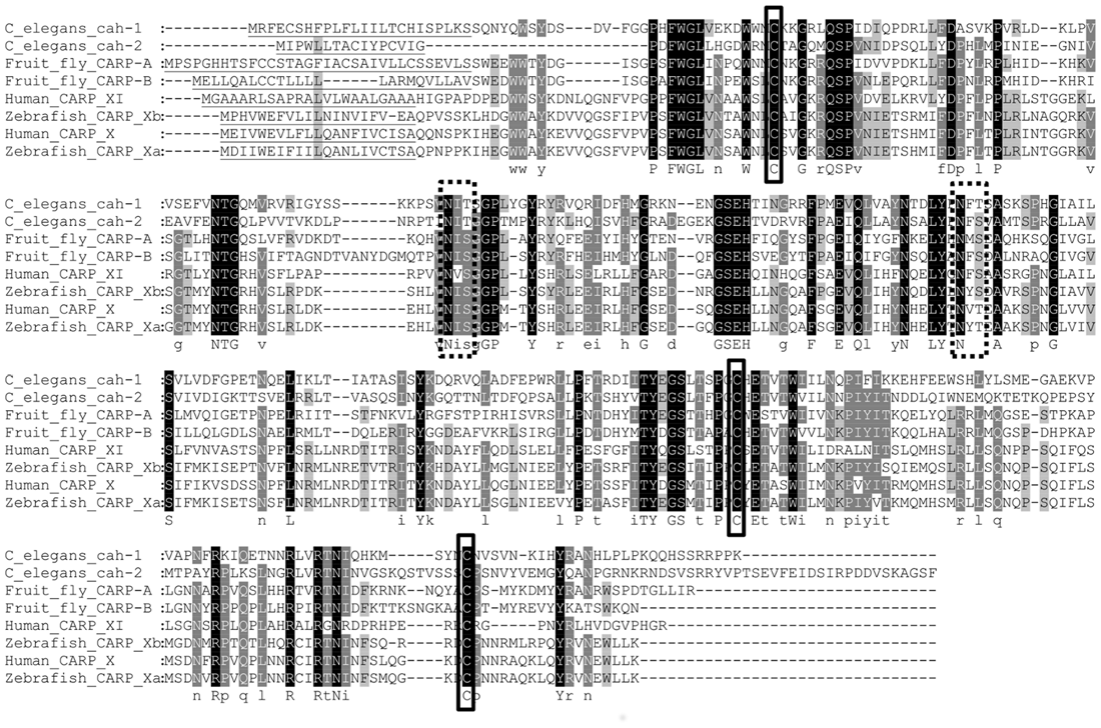
Sequence alignment of human, zebrafish, *C*. *elegans*, and *D*. *melanogaster* CARP X-like proteins. Dashed, broad boxes indicate conserved N-glycosylation sites, and solid, narrow boxes indicate conserved cysteines. Underlining in the N-terminal regions indicates predicted signal peptides.

The third conserved cysteine (C310) occurs in the C-terminal region, which is unique to CARP X-like proteins, and for which no reliable structural template is available. The additional semi-conserved cysteine (C296), seen in CARP X/Xa/Xb but not in CARP XI, would be located near the C terminus of the CA domain. Again, it is noteworthy that the three conserved cysteines are present even in the CARP sequences of nematodes and insects (Fig 4).

Two conserved N-glycosylation motifs are seen in CARP X-like proteins, at N116 and N168 (human CARP X numbering). These positions correspond to non-conserved surface residues, which are not a part of a glycosylation motif (N—not P—S/T—not P) in any other human or zebrafish CA, except for CARP X/XI/Xa/Xb. Likewise, the positions that correspond to the adjacent S/T residues (118 and 170 in human CARP X) are conserved only in CARP X-like proteins. There is a third glycosylation site which is unique to CARP XI sequences, and another one which is unique to CARP X/Xa/Xb.

Fig 4 shows a sequence alignment of CARP X-like proteins of a diverse selection of vertebrates and invertebrates, highlighting the presence of signal peptides, three conserved cysteines, and two conserved glycosylation motifs.

### Zebrafish *ca10a* and *ca10b* genes are strongly expressed in the nervous system and in developing embryos

**Fig 5:**
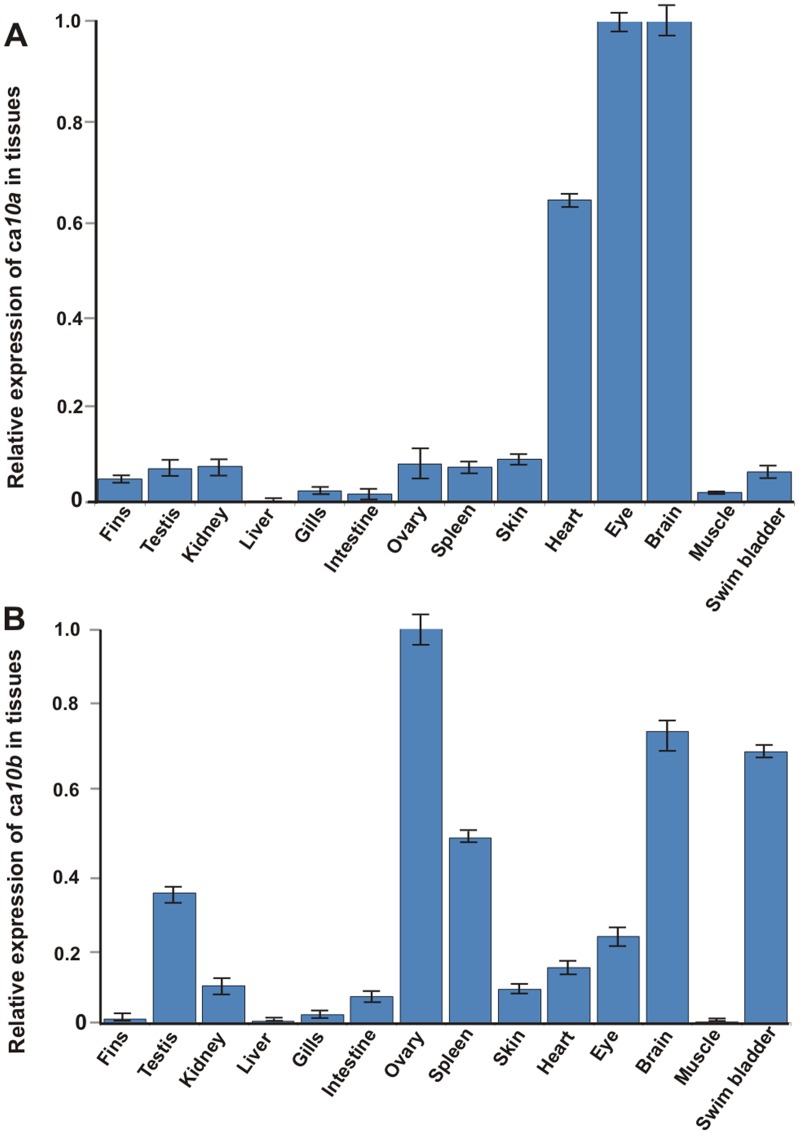
Expression levels of *ca10a* and *ca10b* genes in adult zebrafish tissues. A quantitative analysis of *ca10a* and *ca10b* genes was made for 14 adult zebrafish tissues using RT-qPCR. **A**, expression of *ca10a* in zebrafish tissues; **B**, expression of *ca10b* in zebrafish tissues. The values were normalized to the beta actin control according to the Pfaffl´s equation [34]. The expression of the *ca10a* gene in brain and *ca10b* in the ovary assigned a relative value of 1.

We used RT-qPCR to study the expression of *ca10a* and *ca10b* in adult zebrafish. Expression analysis showed the presence of *ca10a* in most of the studied tissues (Fig 5A). The expression of *ca10a* was highest in the heart, eye, and brain. In addition, *ca10a* was expressed in fins, testis, kidney, gills, intestine, ovary, spleen, skin, muscle, and swim bladder. Interestingly, no *ca10a* expression was detected in the liver (Fig 5A).

The tissue specific expression pattern of the *ca10b* gene showed differences compared to that of *ca10a* (Fig 5B). The highest expression of *ca10b* was found in the ovary, brain, and swim bladder. Expression was also observed in the fins, testis, kidney, gills, intestine, spleen, skin, heart, and eye whereas no signal for *ca10b* was found in the liver or muscle.

**Fig 6:**
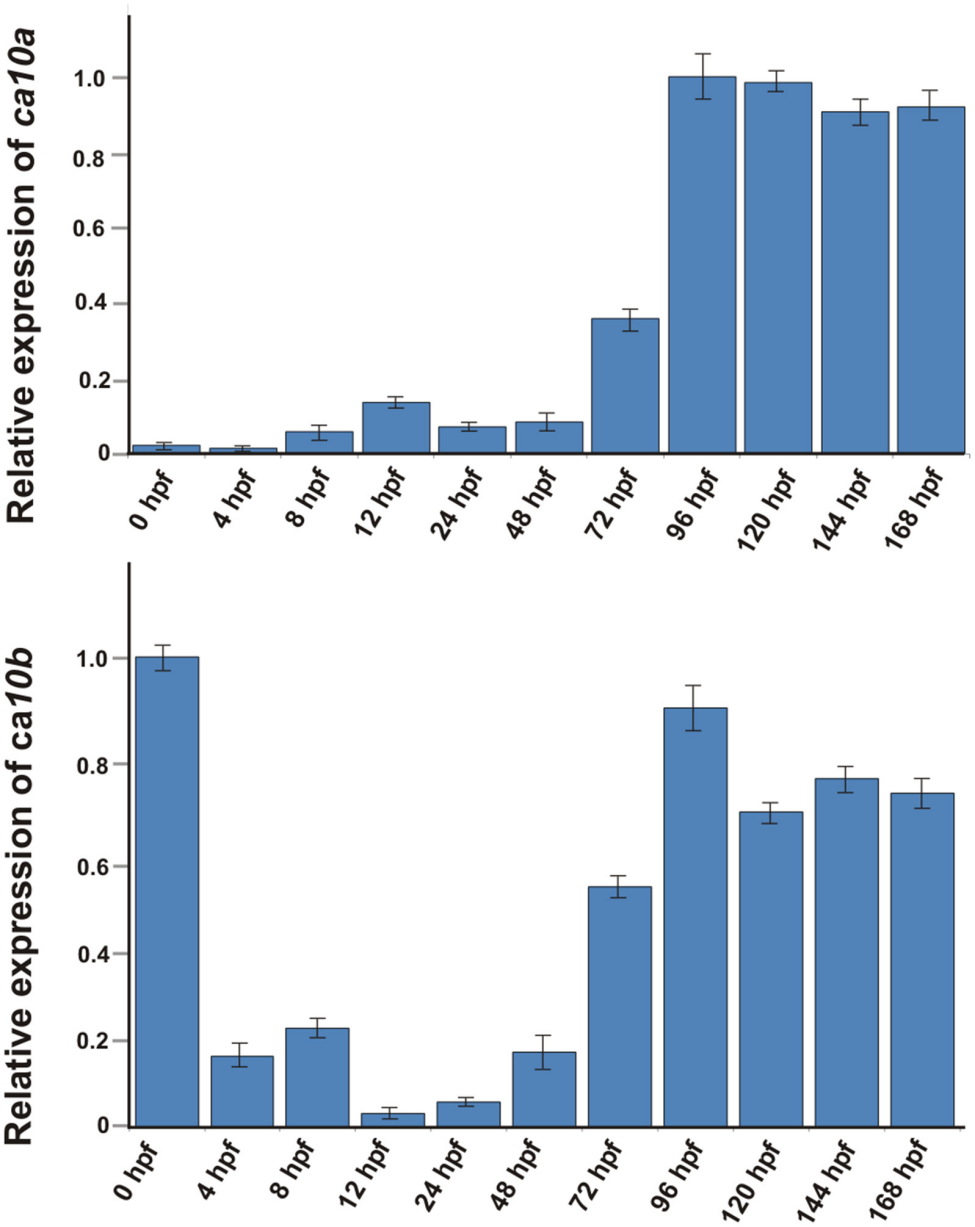
Expression analysis of *ca10a and ca10b* mRNAs during embryonic development. The expression levels of *ca10a and ca10b* were measured from the total mRNA isolated from the developing larvae of 0–168 hpf. **A)** Expression values of *ca10a* mRNA. **B)** Expression values of *ca10b* mRNA. The expression values were normalized to the beta actin control according to the equation of Pfaffl [34]. The expression of *ca10a* gene at 0 hpf and *ca10b* at 96 hpf assigned a relative value of 1.

In addition, we used RT-qPCR to investigate the expression of *ca10a* and *ca10b* during the development of 0–168 hpf (0–7 dpf) zebrafish larvae (Fig 6). Expression of both *ca10a* and *ca10b* were highest at 96 hpf (4 hpf) and remained high thereafter. Low levels of *ca10a* and *ca10b* expression was seen at earlier time points. The expression pattern suggests that the *ca10a* gene product is required throughout embryonic development, especially after 72 hpf (3 dpf). Interestingly, *ca10b mRNA* was present at a very high level at 0 hpf, suggesting maternal origin (Fig 6B) and this is also supported by the high expression in the ovary of the adult fish (Fig 5B). The expression pattern of the *ca10b* gene suggests that this gene plays a role at the beginning of development and is required throughout embryonic development in zebrafish.

### Knockdown of *ca10a* and *ca10b* genes causes developmental abnormalities in zebrafish larvae

**Fig 7:**
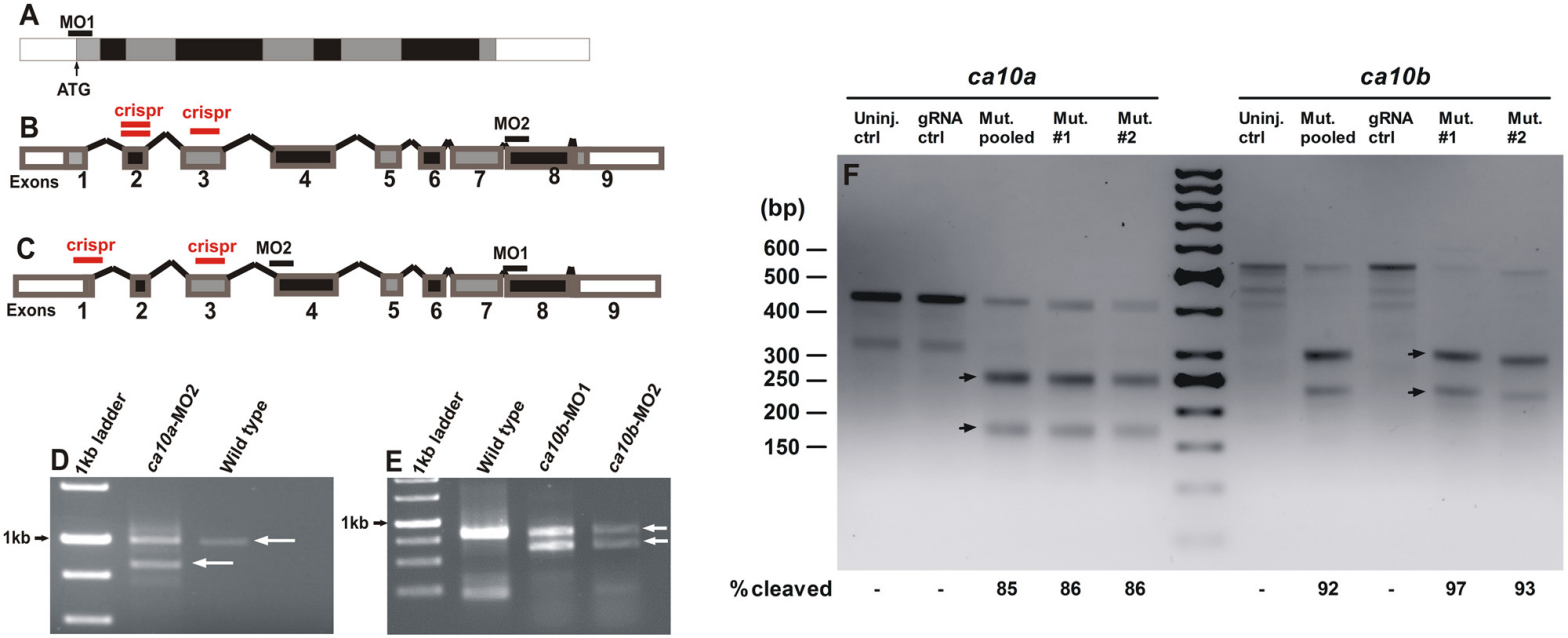
Silencing of *ca10a* and *ca10b* in zebrafish larvae. **A)** Schematic presentation of matured *ca10a* mRNA showing the site of translational blocking with MO1 at the translation start site (arrow). **B)** Schematic structure of unprocessed mRNA for *ca10a* with a target region (horizontal bar) for a splice site blocking morpholino (MO2) which knocks down the exon eight. **C)** Schematic depiction of unprocessed mRNA for *ca10b* and target sites for splice site interfering morpholinos, MO1 and MO2 (black horizontal bars) and gRNA target regions (red horizontal bars). **D)** Gel electrophoresis showing RT-PCR analysis of *ca10a* morphant mRNA injected with MO2. **E)** RT-PCR gel image of *ca10b* morphant zebrafish mRNA injected with MO1 and MO2 targeting different exons. The images show the reduction in the length of the mRNAs (Lane 2 in **D** and Lane 3 and 4 in **E)** compared with wild type mRNAs of *ca10a* and *ca10b* in wild type fish (Lane 3 in **D** and lane 2 **E**). **F)** The efficiency of the CRISPR/Cas9 mediated mutagenesis in zebrafish embryos was evaluated with a T7 endonuclease assay. For both *ca10a* and *ca10b*, uninjected and gRNA control fish are shown and as well as two individual embryos with a mutated target site and a pool of 5–10 mutated embryos. Representative cleaved PCR products of the expected sizes are shown as arrow heads. Cleavage percentage was calculated from the band intensities of each lane.

Evolutionary conservation of *CA10*-like genes, their ubiquitous expression pattern in different tissues, and high mRNA levels during embryonic development suggest a crucial role for CARP X-like proteins in vertebrates [41]. To shed light to the biological significance of CARP X-like proteins in embryogenesis, we silenced zebrafish *ca10a* and *ca10b* genes with MOs (Fig 7A, 7B and 7C).

The translation-blocking antisense MO for *ca10a* (*ca10a*-MO1) targeted a sequence that includes the ATG start codon (from -15 to +10 nucleotides, relative to ATG) and inhibits translation of mRNA. MO2 (*ca10a*-MO2) targeted exon 8, resulting in expression of shorter length *ca10a* mRNA (Fig 7D). Both antisense MOs for the *ca10b* gene (*ca10b*-MO1 and *ca10b*-MO2) resulted in truncated *ca10b* mRNAs as shown in Fig 7E.

**Fig 8:**
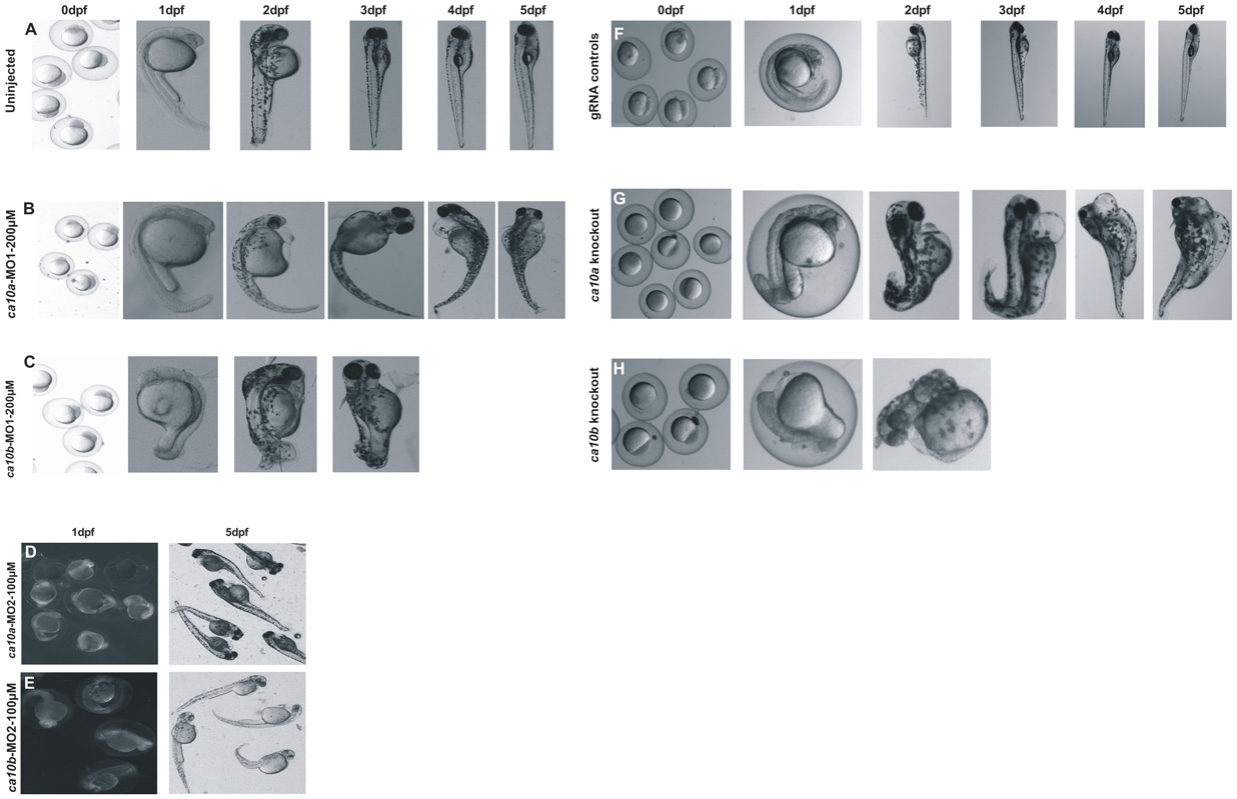
The *ca10a and ca10b* genes play an important role during embryonic development in zebrafish. **Left panel:** Developmental images of *ca10a* and *ca10b* morphant zebrafish injected with two different sets of antisense MOs (MO1 and MO2) over the period of 0 hpf to 5 dpf. **A)** The top row images show 0 hpf embryos and lateral views of 1–5 dpf uninjected zebrafish larvae with a normal development of organs,. **B)** *ca10a* morphant zebrafish injected with 200 μM translational blocking antisense morpholinos (MO1). **C)** Images of *ca10b* morphant zebrafish. **D)** and **E)** Images of *ca10a* and *ca10b* morphants. The images in first panel of **D** and **E** show the 1 dpf larvae with rhodamine fluorescence throughout the larvae (indicating successful MO injection) and second panel (**D** and **E**) shows the 5 dpf larvae with abnormal appearance. **Right panel:** Developmental images of *ca10a* and *ca10b* CRISPR mutated fish and gRNA controls. **F)** The top row images show 0 dpf embryos and lateral views of 1–5 dpf gRNA injected zebrafish larvae (control) with a normal development of organs. **G)** *ca10a* mutated zebrafish injected with gRNA and *cas9* mRNA targeted to exon 3 (target site shown in Fig 7B). **H)** *ca10b* mutated zebrafish injected with gRNA and *cas9* mRNA targeted to exon 1(target site shown in Fig 7C).

**Fig 9:**
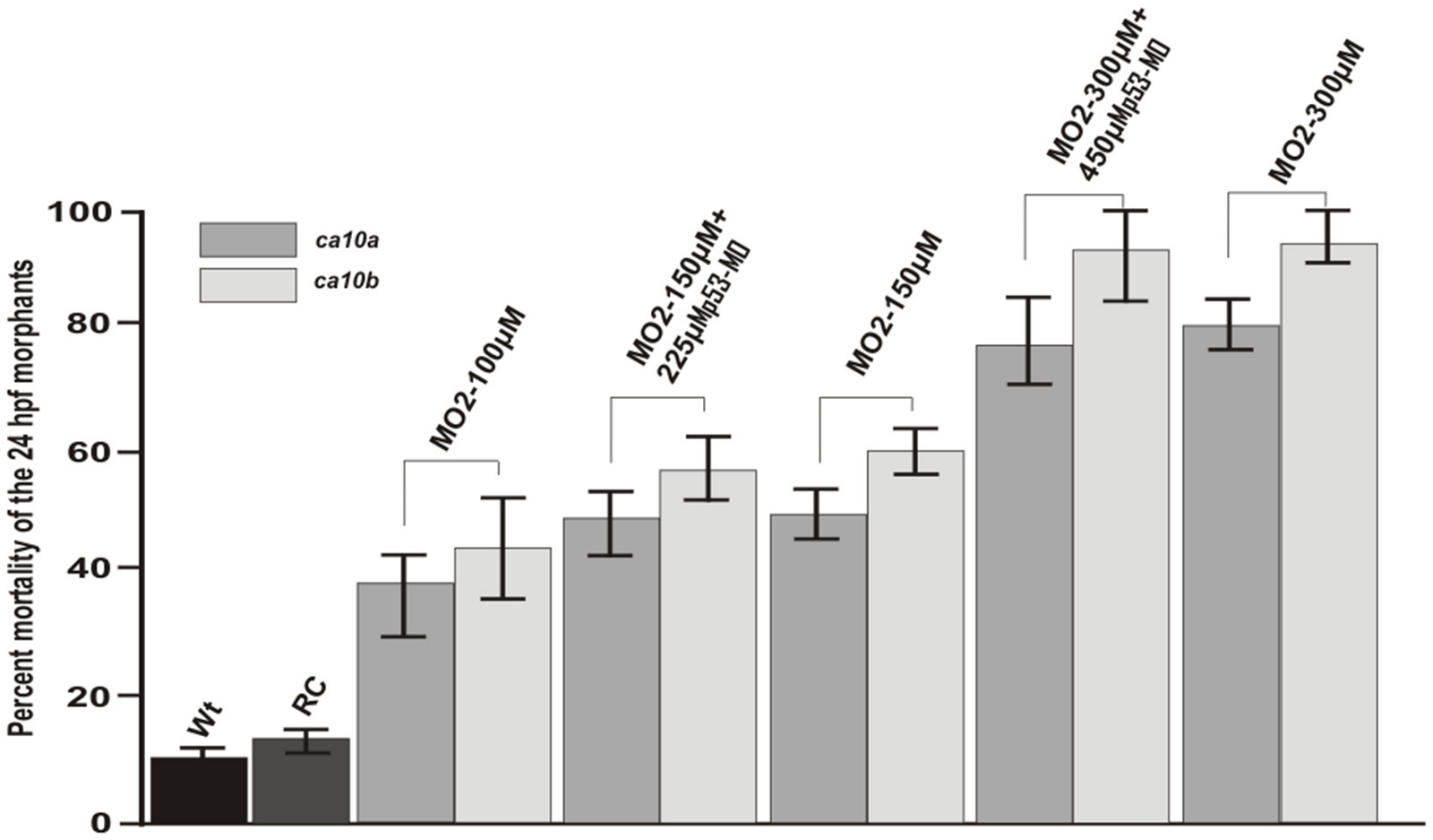
The percent mortality of the morphant larvae at 24 hpf. The mortality rate of *ca10a* and *ca10b* MO injected larvae was significantly higher than that of controls (P< 0.001). Larvae injected with *p53*-MOs along with *ca10a* or *ca10b* -MOs and larvae injected with *ca10a* or *ca10b* -MOs alone did not show significantly different mortality rates.

The 1 dpf zebrafish larvae injected with 200 μM *ca10a*-MO1 showed defects in the head, abnormal body structure, and small eyes with a delayed hatching from the chorion (Fig 8B). As the development progressed, the abnormalities became more prominent; the larvae had long tapering curved tails, curved body structure, pericardial edema, absence of swim bladder, and otolith vesicles (Fig 8B). The embryos injected with *ca10a*-MO2 showed a less severe phenotype compared with the *ca10a*-MO1 injected larvae (Fig 8D). Injection of a higher concentration (300 μM) of *ca10a*-MO led to a more severe phenotype compared to the lower dose with higher mortality in the morphant larvae after 24 hpf (Fig 9 and Table 6). Further increase in the MO concentration (above 300 μM) had lethal effect on the larvae.

**Table 6:** Phenotypes of zebrafish larvae knocked down with different concentrations of *ca10a* and *ca10b* antisense morpholinos.

*ca10a/ca10b*-MO2s (μM)	Normal	Mild	Moderate	Severe	Dead	Total
MO 300	11 (1.8)/ 9(1.5)	16 (2.6) /7 (1.2)	46 (7.6) /37 (6.2)	70 (11.7) /73 (12.2)	457(76.2) /474(94.8)	600
MO 300 + *p53* 300	2 (0)/ 0 (0)	3 (2.0) /0 (0)	4 (9.3) /1 (0.6)	29 (19.3) /8 (5.3)	112 (74.6) /141 (94.0)	150
MO 150	22 (4.4)/ 13 (2.6)	43 (8.6) /32(6.4)	82 (16.4) /47 (9.4)	121 (24.2) /96 (19.2)	232 (46.4) /312 (62.4)	500
MO 150 + *p53* 225	19(3.8)/ 16 (3.2)	38 (7.6) /41 (8.2)	76 (15.2) /32 (6.4)	144 (28,8) /119 (23,8)	223 (44.6) /292 (58.4)	500
MO 100	4 (2.6)/ 2(1.3)	21 (14) /14 (9.3)	27 (18.0) /16 (10.6)	92(61.3) /51 (34)	56 (37.3)/ 67 (44.6)	150
MO 50	102(68)/92(61.3)	27(18.3)/34(22.6)	7(4.6)/5(3.4)	3 (2)/4(2.6)	11(7.3)/15(10)	150
RC MO	422 (84.4)	2 (0.4)	4 (0.8)	11 (2.2)	61 (12.4)	500
Uninjected	446 (89.2)	0 (0)	6 (1.2)	8 (1.6)	40 (8)	500

The phenotypic data was obtained from a minimum of three independent sets of morpholino injections with a minimum of 100–200 zebrafish embryos in each group. The *ca10a* and *ca10b* morphant larvae examined for each group is shown as percentage of total number of larvae studied at 24 hpf. Random control (RC) morpholino injections were made according to the concentration of *ca10a* and *ca10b* MO1 in each set of experiments. Larvae injected with 50 μM concentrations of either *ca10a*-MO2s or *ca10b*-MO2s had a phenotype similar to uninjected larvae but showed abnormal swim patterns (S1 and S2 movies).

The specificities of the MOs were confirmed by analyzing the lengths of *ca10a* and *ca10b* mRNAs from the morphant embryos injected with the splice site blocking morpholinos. Analysis of *ca10a* mRNA from 5 dpf morphant fish injected with *ca10a*-MO2 (designed to knock down one exon in *ca10a* mRNA) showed two bands on the agarose gel, one corresponding to the wild type length mRNA and the other band corresponding to the *ca10a* morphant mRNA (Fig 7D). The analysis of mRNA from 5 dpf morphant zebrafish injected with *ca10b*-MO1 and *ca10b*-MO2 (both targeting different exons in *ca10b* mRNA) showed two short bands in addition to the normal length bands compared with mRNA from wild type fish (Fig 7E). The result of mRNA analysis from the morphant zebrafish confirmed that the defective phenotypes observed in the *ca10a* and *ca10b* morphant fish were due to specific knock down effects of these genes by antisense MOs.

The larvae injected with 200 μM of *ca10b*-MO1 showed severe phenotypic defects as early as 12 hpf (data not shown). The *ca10b*-MO1 morphants at 1 dpf were short and the shape of the body was abnormal. The morphant larvae were very fragile and had a high mortality rate (Figs 8C and 9 and Table 6). They could not survive beyond 3 dpf nor did they hatch properly from the chorion. Injection of *ca10b*-MO2 produced a milder phenotype compared with the larvae injected with *ca10b*-MO1. The *ca10b*-MO2 morphant larvae showed abnormal body structure, smaller head and eye structure, mild pericardial edema, absence of otolith sacs, unutilized yolk sac, and curved tail (Fig 8E).

There was no significant difference between the phenotype defects and mortality rates of larvae injected with *CA-*MOs alone or in combination with *p53*-MOs (Table 6). In addition, the uninjected embryos and embryos injected with RC-MOs showed normal phenotypes and similar survival rates (Fig 9 and Table 6). These results suggest that the phenotypic defects seen in morphant embryos injected with *ca10a* and *ca10b* MOs were most likely due to knockdown of these genes and not p53-dependent off-target effects of antisense MOs, which normally occur in 10–20% of the MO knockdown studies [42].

We validated our results of the *ca10a* and *ca10b* MO knockdown by silencing the respective genes with the CRISPR/Cas9 genome editing method [37,38]. To establish this method, we first targeted *egfp* in a transgenic zebrafish line (*fli1a*:*egfp*) using a previously described gRNA [37], and confirmed mutations in the target region with a T7EI assay (S2 Fig). To silence *ca10a* and *ca10b*, we initially, we designed 3 gRNAs for the *ca10a* gene and 2 gRNAs for the *ca10b* gene, each of which targeted different exons or different locations in the same exon (Fig 7B and 7C). We then compared the cutting efficiency for each gRNA and chose the most efficient gRNAs (*ca10a*-exon 3 gRNA and *ca10b*-exon 1 gRNA) for both genes based on the results of the T7EI assay (Fig 7).

Similar to the MO injected larvae, the phenotype of *ca10b* mutated larvae was severe with high mortality at 1 dpf and larvae did not survive beyond 2 dpf (Fig 8H). The *ca10a* mutated larvae showed a less severe phenotype (Fig 8G) with a lower mortality rate at 1 dpf. Larvae injected with only gRNA showed normal phenotypes compared to uninjected controls. With the T7 assay, we detected a cleavage efficiency of up to 86% for *ca10b* and up to 97% for *ca10a*. For the assay, individual mutated larvae as well as mutated larvae pooled together were analyzed (Fig 7F).

### The *ca10a* and *ca10b* morphant larvae showed morphological changes in the tissues and apoptosis in the head region

The expression pattern of *ca10a* mRNA suggests that CARP Xa protein plays an important role in the brain, eye, and several other tissues. CARP Xb might play some roles in reproduction and brain functions. We studied morphological changes in the larvae injected with 200 μM *ca10a*-MO2 and *ca10b*-MO2 and compared the results with wild type larvae and larvae injected with RC-MOs. We prepared semi-thin (5–10 μm) sections of 5 dpf control larvae, larvae injected with 200 μM *ca10a*-MO2, and larvae injected with 200 μM *ca10b* -MO2 and stained them with Hematoxylin and Eosin. The morphological examination showed gross morphological changes in the head and eye regions of *ca10a* and *ca10b* morphant larvae (results not shown). Similarly, there was a clear difference between the somites of control larvae and those of the *ca10a*-MO and *ca10b*-MO injected morphant larvae.

**Fig 10:**
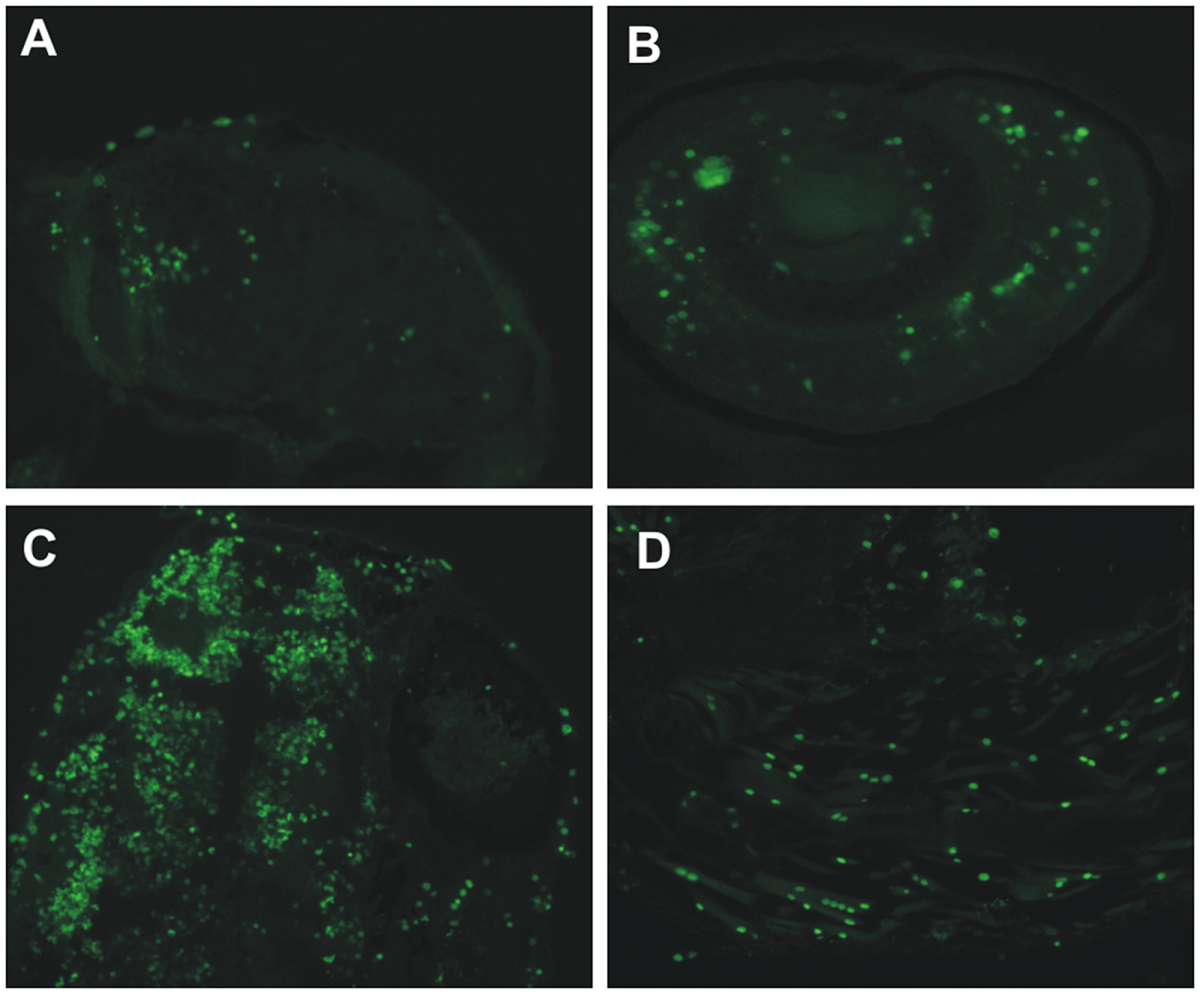
Knockdown of *ca10a* and *ca10b* genes leads to apoptosis in the morphant zebrafish. Results of the TUNEL assay detecting apoptotic cells in 5 dpf morphant embryos. **A)** head region of a *ca10a* morphant; **B)** eye region of a *ca10a* morphant; **C)** head region of a *ca10b* morphant; and **D)** tail regions of a *ca10b* morphant. (Original magnification 100X).

The TUNEL assay on sections of *ca10a*-MO2 injected 5 dpf zebrafish larvae showed apoptotic cells especially in the head and eye regions (Fig 10A and 10B). Similarly, large areas of apoptotic cells were observed in the head region of 5 dpf *ca10b*-*MO2* morphant zebrafish larvae (Fig 10C) and weaker signals were seen in the tail region (Fig 10D). The TUNEL assay did not show any signs of apoptosis in wild-type larvae or larvae injected with RC-MOs (data not shown).

### Human *CA10* and *CA11* mRNAs partially rescue the morphant phenotypes

**Fig 11:**
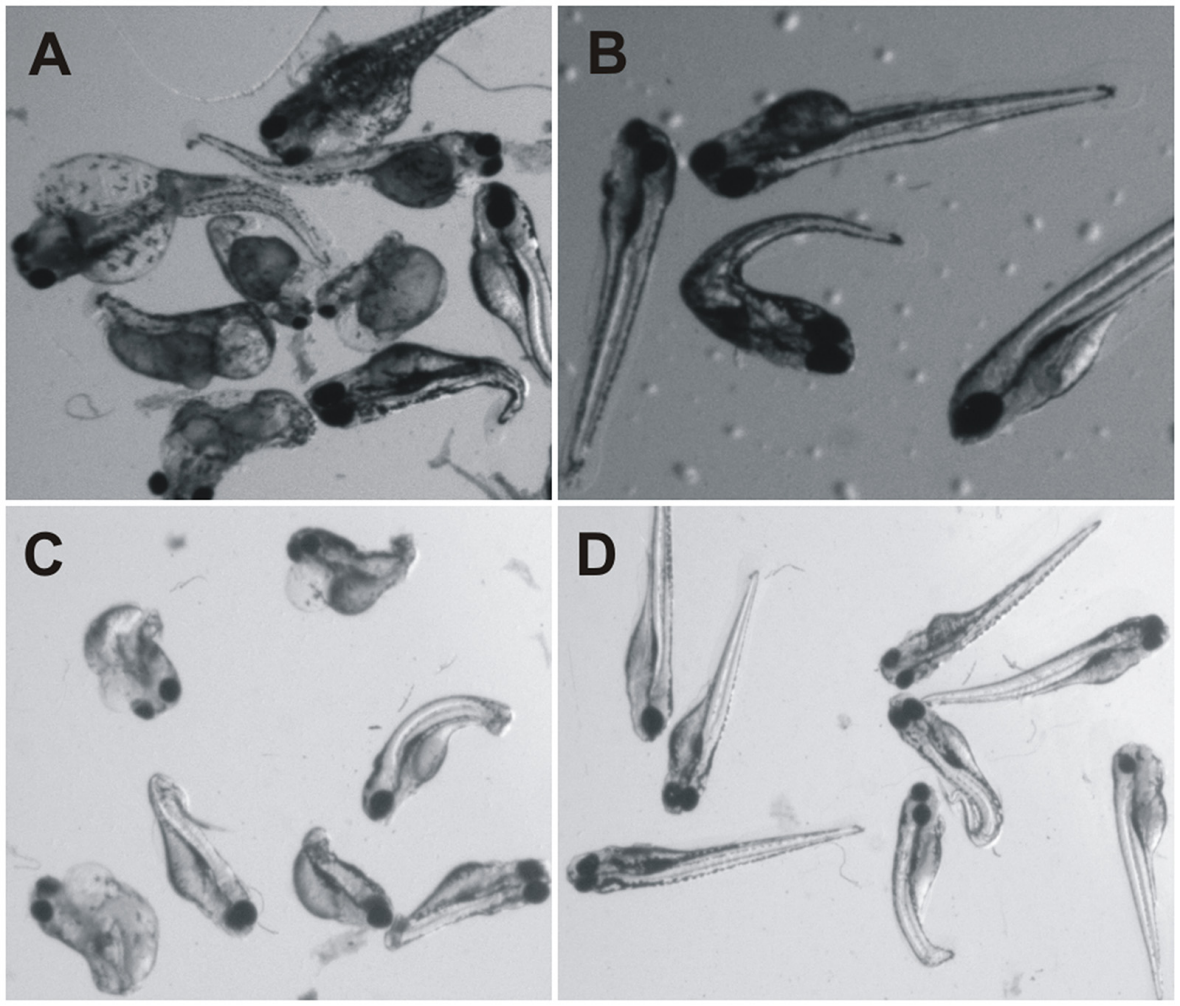
Partial rescue of *ca10a* and *ca10b* zebrafish morphants. **A)** The *ca10a* morphant (5dpf) embryos; **B)** The 5 dpf zebrafish *ca10a* morphant embryos rescued with injection of *CA10* mRNA; **C)** The *ca10b* morphant (5dpf) embryos; **D)** Partially rescued 5dpf embryos with *CA11* mRNA.

To confirm the specificity of the phenotypes produced by antisense MOs, we co-injected the *ca10a* and *ca10b* MOs with capped human mRNAs for *CA10* and *CA11* genes. The morphant fish injected with 80 pg of mRNA per embryo along with 300 μM antisense MOs showed observable improvement in the phenotype of morphant embryos as shown in Fig 11A–11D. The partial rescue of *ca10a* and *ca10b* morphant embryos with the injection of gene-specific human mRNAs also confirmed the specificity of the *ca10a* and *ca10b* antisense MOs used in the study.

### Knockdown of *ca10a* and *ca10b* leads to abnormal movement pattern in zebrafish larvae

**Fig 12:**
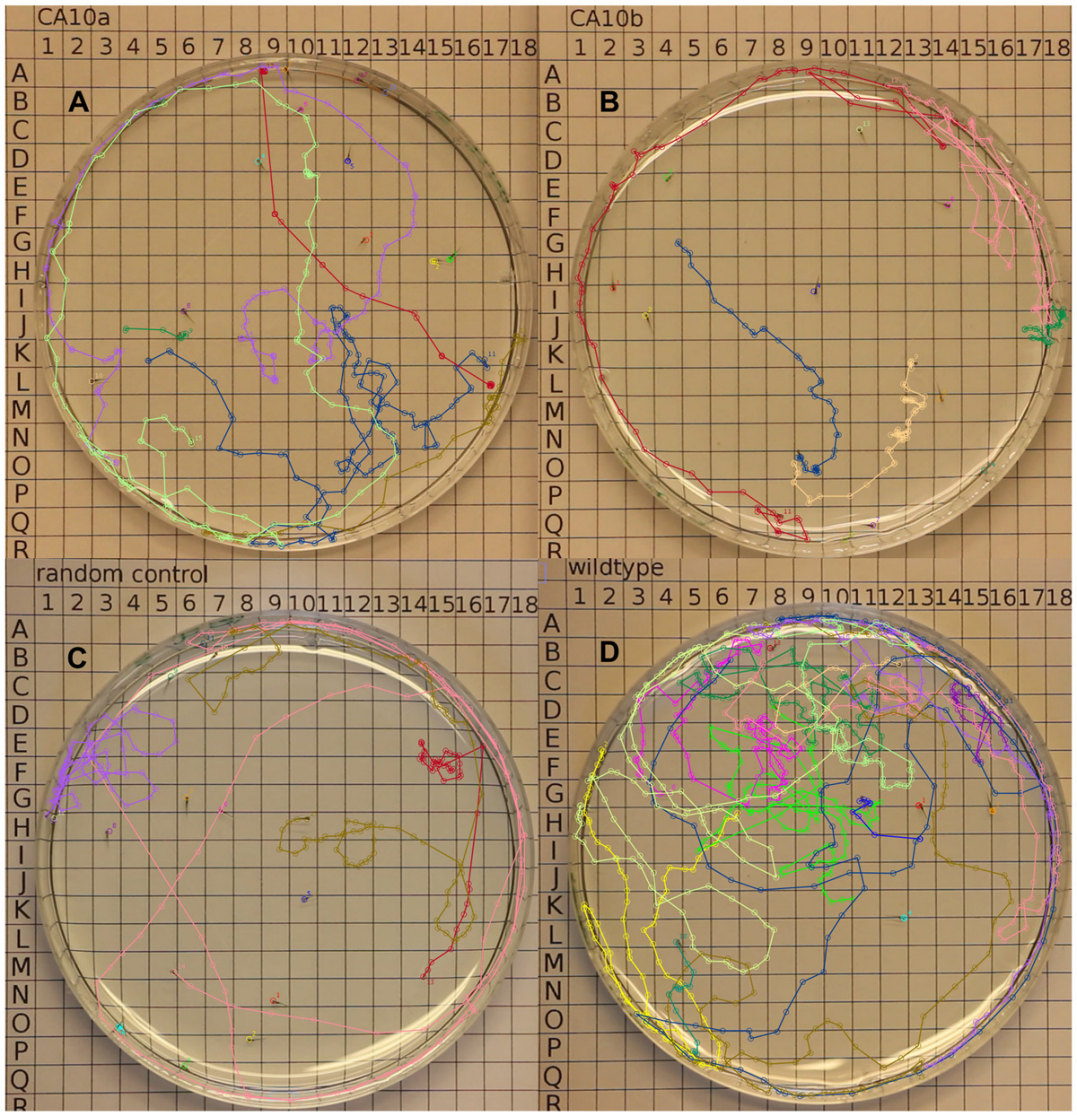
Displacement patterns of the morphant and control fish. Representative displacement trajectories of the movement pattern are shown for **A)** larvae injected with *ca10a*-MO2, **B)** larvae injected with *ca10b-*MO2 **C)** larvae injected with RC-MOs and, **D)** wildtype **(**uninjected) larvae. Groups of 13 to 22 fish were video recorded in 90mm petri dishes over a 2 minute time period. ImageJ and MtrackJ plugin were used to track the paths of all fish [39,40].

**Fig 13:**
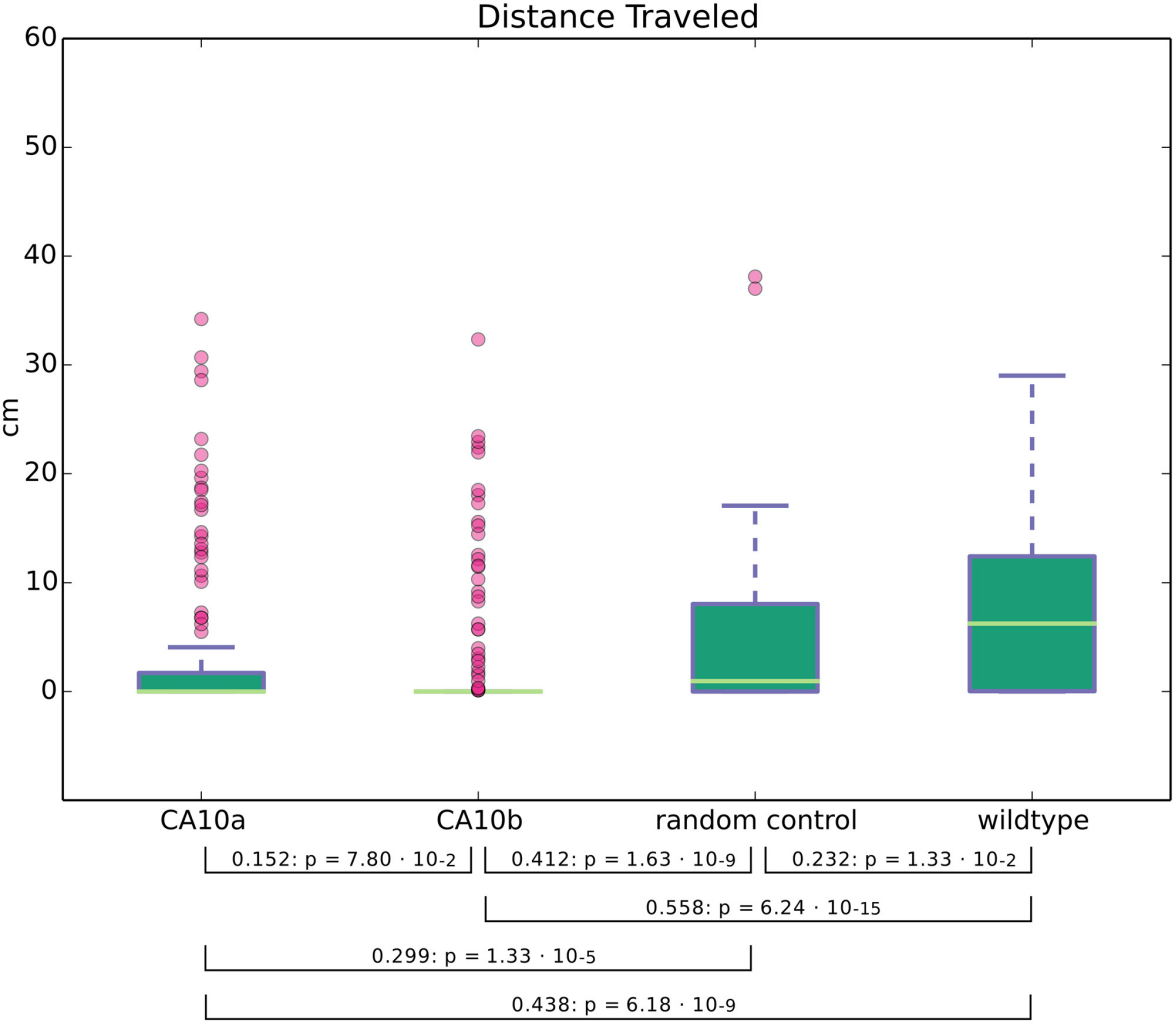
Knockdown of *ca10b* and *ca10b* genes results in abnormal movement pattern in morphant zebrafish larvae. The swim patterns of 5 dpf larvae injected with 100 μM antisense *ca10a-*MO2 or *ca10b-*MO2 were compared to the larvae injected with RC-MO and wildtype (uninjected) controls as described in the methods. (In addition to the swim patterns and body movement videos of 10 larvae from each group created with a Zeiss microscope for close observation as included in the supporting information). Distances traveled by 457 fish in the four groups are presented as boxplots as created with matplotlib [43]. The results of two sample Kolmogorov-Smirnov statistical analyses are included between bracket linked groups.

The expression pattern of *ca10a* and *ca10b* genes suggested that the encoded proteins play important roles in the brain. Therefore, we hypothesized that down regulation of these genes with antisense MOs may lead to changes in behavior or motor coordination, which can be investigated by monitoring the swim pattern of the morphant embryos. It is obvious that the larvae with body and tail abnormalities, induced by high concentration of *ca10a* and *ca10b* antisense morpholinos, would show abnormal swimming behavior. To see any neurological effect on movement pattern as a result of down regulation of these genes, we needed morphant larvae with no obvious phenotypic defects. To test this hypothesis, we injected the larvae with lower concentrations (100 μM) of *ca10a*-MO2 and *ca10b*-MO2. With these concentrations, the 5 dpf larvae showed normal body and tail development with no visible phenotypic defects, similar to RC-MO injected and uninjected larvae (S1–S3 Movies). In the experiment, 4 groups (uninjected, 100 μM *ca10a*-MO injected, 100 μM *ca10b*-MO injected, and RC injected embryos) of larvae were analyzed for movement patterns to observe movement of the body while swimming and traced the movement of the fish (Fig 12). Similarly, we calculated the total distance traveled for all larvae (details in the methods). Two-sample Kolmogorov-Smirnov statistical analyses were performed between each of the groups to determine if they could have been drawn from the same distribution, shown in Fig 13. Both knockdown groups swam significantly smaller distances than the random control and wildtype groups. The distances of the two knockdown groups did not differ significantly. However, fish in the *ca10b* group showed a tendency towards less movement (Fig 13). The distances traveled by fish in the random control group were smaller than those of the wildtype controls. We assume that either the impact of the injection procedure or the MOs themselves account for this difference.

Analysis of the actual swim pattern revealed that the large majority of morphant larvae did not show movement during the 2 minute recording. Those that did, showed slow movements of the tail and tended to remain in the periphery of the petri dish (S2 and S3 Movies and Fig 12A and 12B). Videos obtained through microscopic viewing revealed that *ca10b* morphant larvae had difficulties in balancing the body. The 5 dpf wild type zebrafish controls showed normal swim pattern (S1 Movie, Fig 12D).

## Discussion

The carbonic anhydrase related proteins (CARPs) VIII, X, and XI belong to the α-CA family and are highly conserved across all species, even more so than the enzymatically active CA isoforms [6]. The expression studies of these proteins show that CARPs are predominantly expressed in the CNS [6,10,11,44]. Spontaneously occurring mutations in CARP VIII lead to ataxia and mental retardation in humans and mice [19,20,45]. Recently, we described the function of CARP VIII in zebrafish by knocking the *ca8* gene down using antisense MOs [18]. In addition to aberrations in early embryonic development and brain development, the knockdown of *ca8* led to an ataxic movement pattern, confirming its role in motor coordination [46].

These findings and the exceptionally strong conservation of the *CA10*-like genes prompted us to study the expression of *ca10a* and *ca10b* in zebrafish. The quantitative expression analysis of the *ca10a* gene in developing larvae (0–168 hpf), using RT-qPCR, showed that *ca10a* is expressed throughout the embryonic development and presents the highest level of expression between the period of 96 hpf (4dpf) to 168 hpf (7 dpf). The expression pattern of *ca10b* gene leads us to surmise that initially this gene product is at least partially of maternal origin, with the highest level of *ca10b* mRNA at 0 hpf, consistent with its highest expression in the ovaries of adult zebrafish. The level of *ca10b* mRNA decreased after 0 hpf before rising again at 48 hpf and finally achieving a very high level at 168 hpf. The expression pattern of both genes during development suggests that these genes are required for the embryonic development.

To date, there have been no systematic studies related to the expression of *CA10* and *CA11* genes during the development of a vertebrate animal. There are two studies available related to the expression analysis of CARP X and CARP XI genes during embryonic development [10,11]. In the first study, the expression was studied using RT-PCR method at four different stages of embryonic development in a murine model (E7, E11, E15, and 17) and it was shown that the signal for *CA10* appeared in the middle of gestation (E15) [11]. The signal for *CA11* was seen at an early stage of gestation and became faint as gestation progressed. In the second study, the expression patterns of *CA10* and *CA11* gene products were investigated using immunohistochemistry during five different gestational periods in the human fetal brain [10]. The signal for *CA11* gene product was seen as early as the day 84 of gestation. In contrast, the gene product for *CA10* was not observed until the 121^st^ day of gestation. The signal for CARP X was seen on day 141 of gestation in the neural cell cortex. Both studies on developmental expression suggested that *CA10* appears late in the development and *CA11* is required early in the gestation period, which are in complete agreement with our results and support our hypothesis that these genes play important roles in embryonic development in vertebrates. However, there is still a need for the systematic investigation of expression pattern of *CA10* and *CA11* genes in a mammalian model, such as mouse, during embryogenesis, at both mRNA and protein levels.

In the present study, we have completed a systematic expression analysis of *ca10a* and *ca10b* gene using RT-qPCR in a panel of 14 adult zebrafish tissues. The quantitative analysis of these genes showed that both genes are highly expressed in the brain, confirming the pattern of expression observed in mouse and human. Surprisingly, the zebrafish ovary showed very high expression of the *ca10b* gene, which has not been reported for the genes coding for CARPs in either human or mouse. However, integrated expression data from MediSapiens In Silico Transcriptomics (http://ist.medisapiens.com/) shows a low-level expression of human *CA11* in the ovary. Interestingly, the two CARP X-like proteins in *D*. *melanogaster* have similar expression patterns. CARP-A is exclusively expressed in the brain, whereas CARP-B is predominantly expressed in female reproductive tissues followed by the CNS [8]. It is of interest to note that despite three different histories of duplication, two copies of CARP X-like proteins seem to have adapted to similar roles in mammals, fish, and insects: one exclusively in neural tissues, and the other additionally in ovaries and/or in early embryonic development. Neural expression is seen in *C*. *elegans* as well. Both CARP X-like genes, *cah-1* and *cah-2*, are expressed in several types of neurons [47]. It appears that neural expression of *CA10*-like genes has been conserved since the earliest bilaterian animals [41]. Taken together with the high level of sequence conservation, the universal and ancient history of neural expression suggests some fundamental role for CARP X-like proteins in the brain.

Even though earlier studies have recognized the presence of a signal peptide in CARP X and CARP XI [3,9,12], one later study [10] suggests cytoplasmic localization for CARP X and XI based on histochemical staining, whereas another study by the same authors [11] makes no statements of the subcellular localization. To help resolve this apparent contradiction, we also looked for other features that only have functional significance in secreted, extracellular proteins, and therefore should only be conserved in extracellular proteins: namely disulfides and N-glycosylation motifs. We confirmed that signal peptides are indeed a universal feature in all CARP X-like proteins in seemingly all animal species, including both CARP isoforms in fruit fly and *C*. *elegans*. Surprisingly, we also discovered a conserved pair of cysteines in nearly the same location where all enzymatically active extracellular CA isoforms have a disulfide, and confirmed by protein modelling that these cysteines could form a disulfide. The presence of a third conserved cysteine, which would be unpaired in many CARP proteins, could be a means to form dimers. In addition, we saw two conserved N-glycosylation motifs in positions which are highly variable in other CA isoforms. The disulfide cysteines and the two glycosylation motifs are found in all 61 sequences of our data set, again including both CARP isoforms in fruit fly and *C*. *elegans*. We take these three lines of evidence (conservation of signal peptides, disulfide cysteines, and N-glycosylation motifs) as strong evidence that CARP X, XI, Xa, Xb, and their invertebrate orthologs are all secretory proteins.

Morpholino knockdown is commonly used in zebrafish to study gene specific functions in embryonic development [48]. However, as possible off-target effects possess a major concern, MO experiments need to be validated carefully [49]. To rule out the possibility of off-target effects caused by p53 induced apoptosis [50], we co-injected the *ca10a* and *ca10b* MOs with *p53*-MOs. The co-injection of *p53*-MOs did not show any marked difference on the observed phenotype or on the mortality of the embryos, suggesting that these effects are likely not due to *p53* activation. To exclude other off-target effects, we targeted two different sites of the *ca10a* and *ca10b* genes with MOs and observed that silencing either *ca10a* or *ca10b* with MO1 and MO2 resulted in a similar phenotype between these two MOs. In addition, injection of RC MOs, with concentrations similar to *ca10a* and *ca10b* MOs, did not have any effects on the phenotype of the zebrafish embryos, also supporting gene specific silencing of *ca10a* and *ca10b*. We also showed that the abnormal phenotype of morphant zebrafish could be partially rescued by co-injection of human *CA10* and *CA11* mRNAs. Finally, we validated the results obtained from the MO knockdown of the *ca10a* and *ca10b* genes by silencing these genes using the CRISPR/Cas9 mediated mutagenesis. The phenotypes of the *ca10a* and *ca10b* mutated larvae were similar to those observed in the *ca10a* and *ca10b* morphants.

The members of CARP subfamily (CARP VIII, X and XI) are predominantly expressed in the CNS in both mouse and human, and their high level of sequence conservation suggests that the proteins play important roles there, and missense changes at the sequence level might lead to serious phenotypic consequences. Indeed, this was the case with CARP VIII. Deletion mutations in this gene lead to ataxia and mental retardation in the mice and humans [19,20,45]. The suppression of *ca8* in zebrafish using antisense MOs led to an abnormal swim pattern and reduction in the cerebellar volume; similar to mouse and human where ataxic gait and reduced cerebellar volume are also present [18,51]. In the present study, we examined the behavioral consequences of the *ca10a* and *ca10b* morphant zebrafish larvae. The swim pattern analysis of the larvae at 120 hpf (5 dpf) showed abnormal swim pattern compared with the embryos injected with RC-MOs and uninjected embryos. This finding suggested that even the partial suppression of *ca10a* and *ca10b* genes lead to abnormal motor coordination. TUNEL assay showed neuronal cell death in the head region in the morphant fish injected with *ca10a* and *ca10b* MOs. Interestingly, the recently published report by Hsieh et al. [16] showed that the expression and localization of *CA11* is altered in a human patient with Spinocerebellar ataxia 3, transgenic mice with Machado Joseph Disease, and cultured neuronal cells with a defect in ataxin 3. In fact, all the three CARP transcripts were induced in the neuronal cells with defective ataxin 3 [16]. It has also been suggested that CARP XI, similar to CARP VIII, might interact with inositol 1,4,5-triphospate receptor 1 (ITPR1) and control the release of calcium from the intracellular reserves similar to CARP VIII [16,52], but in light of the evidence that CARP XI would be an extracellular protein, this does not seem a very likely suggestion.

## Conclusions

The present work describes new data on the CARP subfamily of proteins, namely CARP Xa and CARP Xb, in embryonic development in zebrafish. Our work gives insights into the essential roles that *ca10a* and *ca10b* genes play during the embryonic development and how the lack of either of these proteins leads to abnormal motility, similar to zebrafish knocked down for *ca8*. Sequence analysis suggests that all of CARP X, Xa, Xb, XI, and their invertebrate orthologs are secretory proteins, contrary to previously accepted ideas; have a cysteine disulfide; and are potentially dimeric. To our knowledge, this is the first study to reveal that down-regulation of *ca10a* and *ca10b* genes leads to abnormal pattern of movement and also the first one to suggest that these genes are essential for early embryonic development in vertebrates. This study also introduces novel zebrafish models to investigate the mechanisms of CARP Xa and CARP Xb function.

## Supporting Information

S1 Data The data file S1 includes all novel protein and coding sequences based on our improved gene models along with known protein and coding sequences from the Ensembl database. (FASTA)

S1 Fig The modified gRNA design template. (TIF)

S2 Fig Silencing of *egfp* expression in tg(fli1a:egfp) zebrafish embryos using CRISPR/Cas9 mediated mutagenesis. **A)** Fluorescence microscopy was used to analyze the silencing of *egfp* in tg(*fli1a*:*egfp*) zebrafish embryos at 2 dpf. The un-injected control (left) and *egfp* gRNA injected control (middle) express *egfp* (green) in the vascular endothelium. The CRISPR-Cas9 mutated embryo (right) shows less fluorescence due to the disruption of the *egfp* gene. The red channel was used to detect auto-fluorescence. **B)** T7 endonuclease I (T7EI) assay was used to evaluate the *egfp* mutation efficiency in 2-day-old embryos. T7EI treated PCR products of un-injected and *egfp* gRNA injected control fish are shown in comparison to PCR products of two individual embryos and a pooled sample of 5 *egfp* silenced embryos. The full length wild type (WT) *egfp* product (470bp) is marked with an asterix. Arrows indicate the T7E1 cleaved PCR products in the *egfp* mutated embryos.
(TIFF)

S1 Movie The movie shows the swim pattern of wild type 5dpf zebrafish. (MPG)

S2 Movie The movie shows the swim pattern of *ca10a* morphant 5dpf zebrafish. (MPG)

S3 Movie The movie shows the swim pattern of *ca10b* morphant 5dpf zebrafish. (MPG)

S1 Table CARP X-like sequences from different species. The table shows the details of the sequences used in the phylogenetic tree.
(XLSX)
