# Supplementary figures and images for: Inactivation of ca10a and ca10b Genes Leads to Abnormal Embryonic Development and Alters Movement Pattern in Zebrafish
Source: PLoS One. 2015 Jul 28;10(7):e0134263. doi: 10.1371/journal.pone.0134263 (PMC4539348; doi:10.1371/journal.pone.0134263)

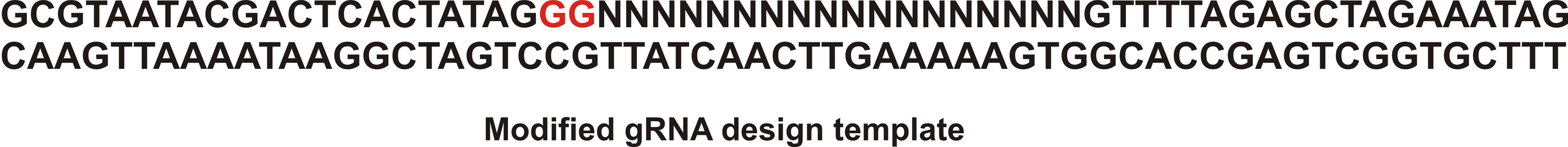

Supplement: S1 Fig — (TIF) [file pone.0134263.s002.tif]

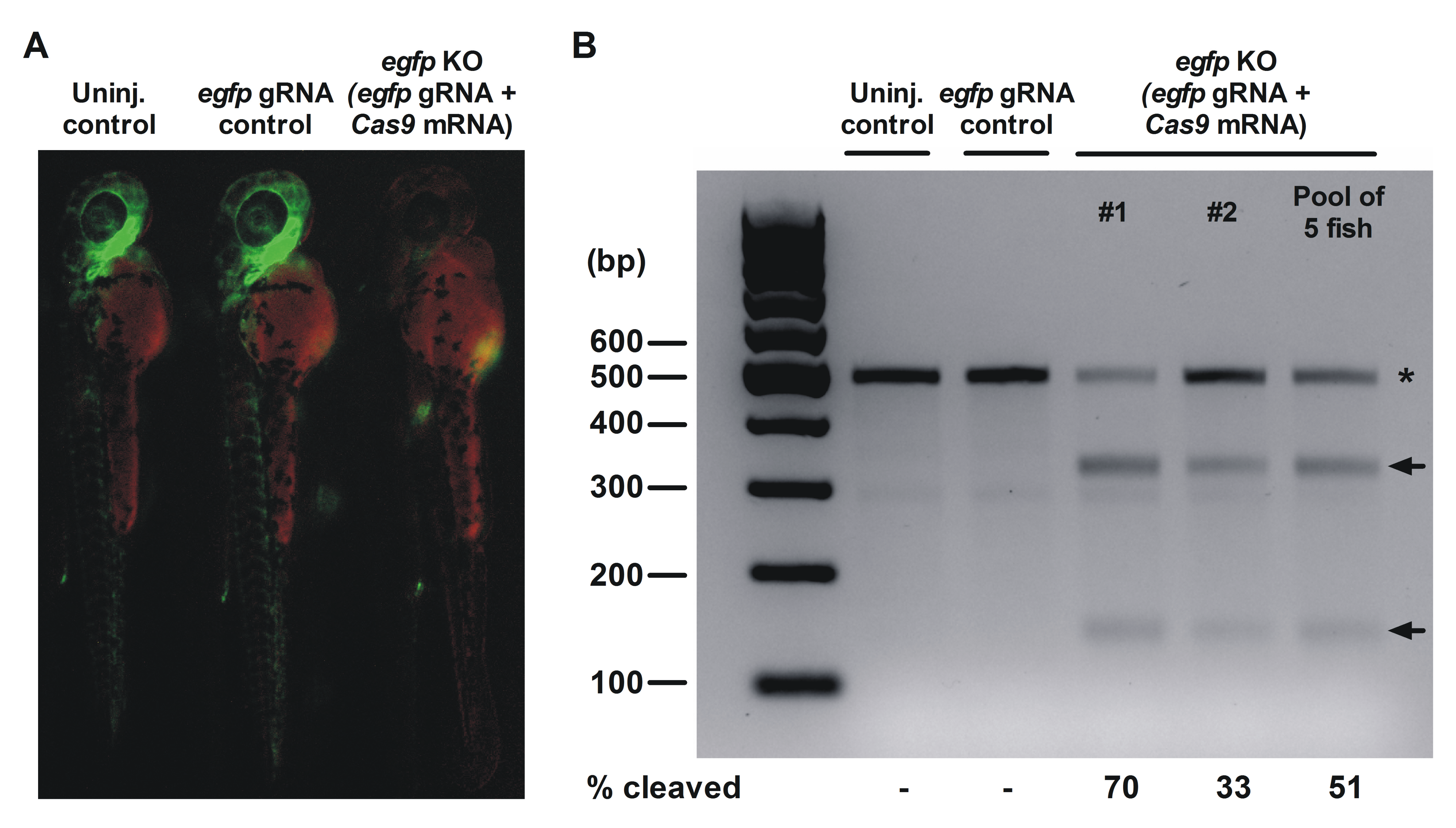

Supplement: S2 Fig — A) Fluorescence microscopy was used to analyze the silencing of egfp in tg(fli1a:egfp) zebrafish embryos at 2 dpf. The un-injected control (left) and egfp gRNA injected control (middle) express egfp (green) in the vascular endothelium. The CRISPR-Cas9 mutated embryo (right) shows less fluorescence due to the disruption of the egfp gene. The red channel was used to detect auto-fluorescence. B) T7 endonuclease I (T7EI) assay was used to evaluate the egfp mutation efficiency in 2-day-old embryos. T7EI treated PCR products of un-injected and egfp gRNA injected control fish are shown in comparison to PCR products of two individual embryos and a pooled sample of 5 egfp silenced embryos. The full length wild type (WT) egfp product (470bp) is marked with an asterix. Arrows indicate the T7E1 cleaved PCR products in the egfp mutated embryos. (TIFF) [file pone.0134263.s003.tiff]
